# Group A streptococcal PerR coordinates iron and zinc homeostasis through Dpr, aiding in bacterial fitness during endothelial cell infection

**DOI:** 10.1128/msystems.01636-25

**Published:** 2026-01-26

**Authors:** Marcia Shu-Wei Su, Chia-Jung Lee, Yi-Lin Cheng, Wei-Jiun Tsai, Chuan Chiang-Ni, Kai-Yu Wang, Yi-Chun Hsieh, Chen-Chieh Liao, Jiunn-Jong Wu

**Affiliations:** 1 Department of Medical Laboratory Science and Biotechnology, College of Medical and Health Sciences, Asia University 63267, Taichung, Taiwan; 2 Department of Biotechnology and Laboratory Science in Medicine, College of Biomedical Science and Engineering, National Yang Ming Chiao Tung University 34914, Taipei, Taiwan; 3 Institute of Basic Medical Sciences, College of Medicine, National Cheng Kung University 38026, Tainan, Taiwan; 4 Department of Medical Laboratory Science and Biotechnology, College of Medicine, National Cheng Kung University 38026, Tainan, Taiwan; 5 Department of Microbiology and Immunology, College of Medicine, National Cheng Kung University 38026, Tainan, Taiwan; 6 Center of Infectious Disease and Signaling Research, College of Medicine, National Cheng Kung University 38026, Tainan, Taiwan; 7 Department of Microbiology and Immunology, Chang Gung University 56081, Taoyuan, Taiwan; 8 Department of Life Sciences, College of Bioscience and Biotechnology, National Cheng Kung University 535291, Tainan, Taiwan; 9 Department of Medical Research, China Medical University Hospital, China Medical University 665203, Taichung, Taiwan; University of Wisconsin-Madison, Madison, Wisconsin, USA

**Keywords:** group A *Streptococcus*, PerR, Dpr, ROS, zinc homeostasis, human endothelial cells

## Abstract

*Streptococcus pyogenes* (group A *Streptococcus*, GAS) causes various clinical complications and invasive diseases. Our previous studies have shown that GAS survives inside endothelial cells due to the insufficient acidification of lysosomes, which fuse with reactive oxygen species (ROS)-induced phagosomes of LC3-associated phagocytosis. For catalase-deficient peroxide-producing GAS to survive in hosts, GAS uses a peroxide response regulator (PerR) to modulate ROS-induced oxidative stress and metal ion regulation. However, it remains unclear whether PerR regulates zinc homeostasis during infections. We generated the GAS Δ*perR* isogenic mutant and conducted dual RNA-seq analysis, an endothelial cell infection model, computational predictions, and phenotypic characterization to demonstrate the protective role of PerR in GAS survival in endothelial cells. The Δ*perR* mutant’s vulnerability to zinc deprivation demonstrated that PerR coordinates iron and zinc homeostasis, likely using PmtA’s iron efflux, iron and zinc-chelating ferritin-like Dpr, the AdcR regulon (*adcA*, *adcAII*, and *phtD*), and zinc efflux (*czcD*). We also demonstrated that the wild-type strain and Δ*perR* mutant encounter zinc restriction inside the phagolysosome GAS-containing vacuoles of endothelial cells. This host zinc starvation severely reduces the survival of the Δ*perR* mutant. These results suggest that the PerR-mediated iron and zinc modulation through Dpr is more important than had been previously thought. Consequently, PerR enhances GAS fitness during its invasions of human endothelial cells.

**IMPORTANCE:**

Our study combines dual RNA-seq analysis, an endothelial cell infection model, computational predictions, and phenotypic characterization to discover the impact of group A *Streptococcus* (GAS) PerR on the coordination of iron and zinc homeostasis during infection. We found that PmtA’s iron efflux, iron and zinc-chelating ferritin-like Dpr, the AdcR regulon, and zinc efflux are delicately modulated by PerR. We also determined that zinc limitation inside the phagolysosome GAS-containing vacuoles of endothelial cells causes host zinc starvation, resulting in reduced survival of the Δ*perR* mutant. Consequently, PerR enhances GAS fitness through Dpr during its invasions of human endothelial cells. Our novel findings offer new insights into how GAS combats iron-mediated oxidative stress and zinc homeostasis that may help develop new anti-GAS treatments.

## INTRODUCTION

Streptococcal infections caused by human obligate group A *Streptococcus* (GAS; *Streptococcus pyogenes*) encompass a wide spectrum of diseases with various levels of severity. Patients with non-symptomatic or mild pharyngitis and impetigo may transmit GAS through droplets and physical contact. If not treated properly with antibiotics, mild GAS infections may sometimes worsen and become severe invasive GAS (iGAS) diseases, such as necrotizing fasciitis and streptococcal toxic shock syndrome, both of which have high mortality rates (1, 2). It has been shown that GAS possesses a multitude of defense mechanisms for immune evasion, enabling it to achieve a successful infection (1, 3).

For catalase-deficient, peroxide-producing GAS to survive in host cells, GAS needs to modulate the reactive oxygen species (ROS)-induced oxidative stress from innate immune responses and metal ion regulation of host nutritional immunity. It has been recently shown how effectively nutritional immunity allows the host to sequestrate and intoxicate trace elements (metal ions) to defend against bacteria at the sites of infection (4–6). Metal ions are essential for promoting enzymatic activity, stabilizing protein structure, and facilitating cellular processes. In humans, iron and zinc are the first and second most abundant trace elements in cells (7). Studies have shown that the homeostasis of iron and zinc plays a crucial role in the defense of hosts against bacterial infections (6, 8–11). However, excess metals are also dangerous as they may generate redox-active agents or create dysfunctional proteins by forming non-cognate metal binding of metalloproteins (5). Unfortunately, studies that examine how GAS combats the host’s innate immune responses and metal ion modulation during infections have rarely focused on the investigation of the mechanisms used in endothelial cells, which are the last barrier against local infections before dissemination.

In our previous work, we have discovered that iGAS proliferates inside the less acidified LC3-associated phagosomes (LAPosomes), which are derived from ROS-associated LAP in human endothelial cells (12, 13). However, it is still unclear how GAS overcomes the ROS-induced oxidative stress and the metal stress of immune responses during its invasions of human endothelial cells. Several studies have shown that the GAS peroxide response regulator (PerR) is involved in the modulation of oxidative stress and metal ion homeostasis. The regulon of PerR comprises iron-related ROS detoxification genes, and some of these genes possess a Per box in their promoter regions (14, 15). These genes encode the Dps-like peroxide resistance and ferritin-like protein (Dpr), the iron efflux heavy metal translocating P_1B-4_-type ATPase (PmtA), the thiol-specific alkyl hydroperoxide reductase (AhpC), the NADH oxidase (Nox1)/alkyl hydroperoxide reductase (AhpF), the glutathione peroxidase (GpoA), and the superoxide dismutase A (SodA) (14, 16–23). The PerR regulon also contains zinc homeostasis genes. Some of them are included in the regulon of GAS AdcR (adhesion competence regulator in the zinc-sensing MarR family) with AdcR motifs in their promoters for zinc acquisition (24–26). Moreover, a cation diffusion facilitator family zinc exporter (CzcD) has also been found to be part of the PerR regulon (25–30).

The aforementioned studies on the transcriptome analysis of *perR* mutants were conducted using only a cDNA microarray with GAS grown in growth media, but not cell infection or animal models (15, 25, 31). Furthermore, it remains unclear whether PerR regulates certain zinc homeostasis genes. Therefore, in this study, we attempted to investigate the molecular mechanisms of PerR’s contribution to iron-related ROS and GAS pathogenesis during infections, with special attention to GAS’s defense against innate immune response and metal ion (iron and zinc) homeostasis. We hypothesized that GAS expresses the stress regulator PerR and then initiates a regulatory network in PerR to protect it from the clearance of human endothelial cells. These results suggest that the Δ*perR* mutant’s vulnerability to zinc deprivation demonstrates that PerR coordinates iron and zinc homeostasis, likely by using PmtA and Dpr proteins, and thus enhances GAS fitness during its invasions of human endothelial cells.

## MATERIALS AND METHODS

### Bacterial strains and growth conditions

GAS strain NZ131 (serotype M49; ATCC BAA-1633) was inoculated on tryptic soy agar containing either 0.5% yeast extract (TSBY) (BD Biosciences, San Jose, USA) or 5% sheep blood (Creative Life Science, New Taipei City, Taiwan) at 37°C in 5% CO_2_ for 20 h, unless otherwise indicated. We cultured GAS single colonies in TSBY broth overnight, then refreshed at a 1:40 dilution until they reached an OD_600_ of 0.5–0.6 (mid-logarithmic phase) at 37°C in 5% CO_2_. Antibiotics kanamycin (Km, 100 µg/mL) and erythromycin (Ery, 5 µg/mL) were added to the media when growing GAS ∆*perR* mutant (Km^r^) and ∆*perR::perR* complemented mutant (Km^r^Ery^r^).

### Construction of GAS ∆*perR*, ∆*dpr*, ∆*perR*::*perR* complemented, ∆*dpr*(pDpr) complemented, and WT(pDpr) strains

To construct a ∆*perR* deletion mutant, we employed homologous recombination and gene replacement approaches (32). A 1,594-bp fragment of *perR,* along with its upstream and downstream flanking sequences, was amplified from the genomic DNA (gDNA) of NZ131 wild-type (WT) strain using primer pair sets perR(spy49_0165)-PCR-1-*Sma*I and perR(spy49_0165)-PCR-2-*Bam*HI (Table S1). The amplicon was ligated to *Sma*I/*Bam*HI-cleaved pSF152 to generate pSF152::*perR*. Next, we carried out an in-frame deletion of *perR* using inverse PCR with primers perR(spy49_0165)-mut-1d*-*Pst*I and perR(spy49_0165)-mut-2-*Xho*I (Table S1), followed by ligation with a blunt-end kanamycin (Km) resistance cassette. The resultant pSF152::*perR*::Km was electro-transformed to the GAS WT strain. After homologous recombination, the Km-resistant transformants were selected and inoculated for gDNA isolation. The ∆*perR* mutant with the deleted *perR* gene was validated using PCR, Sanger sequencing analysis, Southern blotting analysis, and whole-genome sequencing analysis (accession: PRJNA1357628).

Generation of the ∆*dpr* mutant was conducted in a different approach using a temperature-sensitive shuttle vector (pCN143). In brief, the upstream fragment (935 bp) of *dpr* was amplified using dpr_del_1_*Bam*HI and dpr_del_2*_*Stu*I, whereas the downstream fragment (1,358 bp) was generated using dpr_del_3*_*Stu*I and dpr_del_4_*Bam*HI (Table S1). These DNA fragments were *Bam*HI/*Stu*I cleaved, followed by ligation into the *Bam*HI-cleaved pCN143 vector. The resultant pCN143-dpr-KO-reverse#101 obtained an in-frame deletion of *dpr* without an antibiotic resistance cassette. After electro-transformation, temperature shifts, and curing test, the ∆*dpr* mutant was selected and verified using PCR (primers: dpr_del_1_*Bam*HI and dpr_del_4_*Bam*HI), Sanger sequencing analysis (primers: prepilin_peptidase_qPCR_F1 and YqgQ_qPCR-R1), and complementary DNA-quantitative PCR (cDNA-qPCR) analysis (dpr-qPCR-1 and 2; prepilin_peptidase_qPCR_F1 and R1; YqgQ_qPCR-F1 and R1) (Table S1) to confirm *dpr* deletion and polarity.

The complemented ∆*perR::perR* mutant was constructed using an in *trans* approach. In brief, a 2,377-bp fragment of *perR* with its upstream flanking sequence was amplified from the gDNA of NZ131 WT strain using primer pair sets perR-comple-2-*Sal*I and perR-comple-13-*Bam*HI (Table S1). The amplicon was restriction enzyme-digested using *Bam*HI and *Sal*I, followed by ligation to *Bam*HI/*Sal*I-cleaved pTRKL2 shuttle vector to generate pTRKL2(*perR*).

We electro-transformed pTRKL2(*perR*) to the ∆*perR* mutant strain to select Km^r^Ery^r^ ∆*perR::perR* transformants. We applied a similar strategy to generate the ∆*dpr*(pDpr) complemented strain. Using primers dpr_comple_F_*Bam*HI and dpr_comple_R_*Sal*I (Table S1), we amplified a 1,011-bp fragment of *dpr,* including its promoter region. The *Bam*HI/*Sal*I-cleaved amplicon was then ligated to the *Bam*HI/*Sal*I-cleaved pTRKL2. After the transformation, the resultant pDpr (Ery^r^) was confirmed using PCR and Sanger sequencing. The pDpr was introduced to the ∆*dpr* strain to obtain the ∆*dpr*(pDpr)-complemented strain and to the WT strain to generate an overexpressed Dpr in the WT background that was also known as WT(pDpr).

### Hydrogen peroxide challenge *in vitro*

Twenty-five milliliters of GAS strains (WT, ∆*perR*, and ∆*perR::perR*) was refreshed in TSBY broth with appropriate antibiotics to reach the mid-logarithmic phase and divided into 5 mL of GAS culture per tube. The GAS cells were pelleted and resuspended in 5 mL of freshly prepared H_2_O_2_ solution (0, 0.3, and 3 mM in M200 medium) for the challenge at 37°C for 30 min. After serial dilutions, GAS was plated on TSBY, TSBY-Km, or TSBY-KmEry agar plates for 20-h incubation. The GAS survival rate after the challenge was calculated as the percentage of the H_2_O_2_-treated colony-forming unit (CFU) count/0 mM H_2_O_2_ (M200 only)-control CFU count for each GAS strain.

### Human microvascular endothelial cell line-1 cell inoculation and infection conditions

Human microvascular endothelial cell line-1 (HMEC-1) cells (Centers for Disease Control and Prevention, Atlanta, GA, USA) were cultured in endothelial cell growth medium M200 (M200-500; Gibco, Thermo Fisher Scientific, Waltham, USA) with the addition of 10% fetal bovine serum (#SH30396.03; Cytiva, Thermo Fisher Scientific) and 1% low serum growth supplement (S00310, Gibco). HMEC-1 cells were incubated at 37°C in 5% CO_2_ and passaged when cells reached 80% confluency.

For the GAS infection model, HMEC-1 cells were seeded in either a 10 cm dish (1 × 10^6^ cells), 6-well (3 × 10^5^ cells), or 24-well (1 × 10^5^ cells) culture plates overnight. The next day, the refreshed GAS at the mid-logarithmic phase was collected, washed, and resuspended in the M200 medium. We then added the freshly prepared GAS in the seeded cell wells at a multiplicity of infection (MOI) of 50 for infection. After 30 min post-infection, the extracellular GAS in the medium was discarded, and the cells were washed twice with PBS buffer. Next, the cells were inoculated in M200 medium containing the antibiotic gentamicin (125 µg/mL) for another half hour and 4.5 h, counted as 1 and 5 h post-infection, respectively.

For GAS survival in HMEC-1 cells, at 1 and 5 h post-infection, we applied 1 mL of sterile distilled water to the cells of 24-well plates as hypotonic lysis for 1 min at room temperature (RT). The resultant solution underwent serial dilutions and was plated on TSBY or TSBY-Km agar plates to verify live GAS CFUs.

### Confocal immunofluorescence microscopic analysis

HMEC-1 cells were seeded on the coverslips in 24-well plates (1 × 10^5^ or 6 × 10^4^ cells per well) for monitoring GAS invasion at 1 and 5 h post-infection. In brief, 200 nM LysoTracker (L7528, red DND-99, acidic pH indicator; Thermo Fisher Scientific) or 2.5 µM FluoZin-3 AM (#F24195, zinc staining dye; Invitrogen, Thermo Fisher Scientific) was applied to the HMEC-1 cells and incubated in the dark for 1 h at 37°C prior to GAS infection endpoint. Once it reached the infection time point, we applied PBS buffer to wash HMEC-1 cells and used 4% paraformaldehyde as fixation solution for 20 min at RT. Afterward, the fixed cells were washed in PBS buffer and treated in the Blocking and Permeabilization buffer (1% BSA, 0.1% saponin, and 0.05% NaN_3_ in PBS buffer) for 1 h at RT. The primary antibodies (1:200 dilution), anti-LC3 (M152-3, mouse; MBL, Japan), anti-LAMP-1 (9091CS, rabbit; Cell Signaling, USA), or anti-GAS (GTX36330; GeneTex, Taiwan) were then added to the cells on the coverslips and incubated overnight at 4°C. The next day, we washed the cells using PBS buffer and stained them with Alexa Fluor-conjugated secondary antibodies (1:500 dilution) (Alexa Fluor 488; Alexa Fluor 594, #A21207, Donkey anti-Rabbit IgG; Alexa Fluor 647, #A31571, Donkey anti-Mouse IgG; Thermo Fisher Scientific) for 1 h at RT. After PBS washing, 4′,6-diamidino-2-phenylindole (1:5,000 dilution) (DAPI; 508741, Merck, Germany) was applied for DNA staining for 20 min at RT. Finally, the coverslips were PBS washed and mounted using ProLong Diamond Antifade Mountant (#P36970, Invitrogen) onto glass slides for microscopic analysis. We used a confocal microscope (OLYMPUS FV3000) equipped with a 405 nm (cyan)/488 nm (green)/640 nm (magenta) filter set to capture cell images, which were further analyzed using MetaMorph imaging software and the ImageJ-win64.

### GAS growth curve analysis

Overnight-grown GAS strains (WT, ∆*perR*, and ∆*perR::perR*) were diluted in TSBY broth with or without supplements to have a final starting cell density of OD_600_= 0.05 in 200 µL (~3 × 10^6^ CFU in total) in 96-well plates. GAS growth (OD_600_) was measured every 30 min at 37°C using TECAN Infinite 200 Pro (TECAN, Mannedorf, Switzerland). The supplements added to TSBY broth included H_2_O_2_ (0.3 and 3 mM) (Panreac, Italia), zinc sulfate (ZnSO_4_; 0.5, 1, and 2 mM) (SERVA, USA), and zinc chelator Tetrakis-(2-pyridylmethyl)ethylenediamine (TPEN; 25, 35, and 50 µM) (Abcam, UK). For the growth curve of WT, WT(pDpr), ∆*dpr*, ∆*dpr*(pDpr), and ∆*perR* mutants inoculated in the TSBY broth or supplemented with 0.8 mM ZnSO_4_, we either measured manually every hour or used an ODBox-C (TAITEC Corporation, Koshigaya City, Saitama, Japan) every 30 min to detect GAS density at OD_600_ at 37°C for at least 9 h.

### RNA isolation

For polar effect analysis of the ∆*perR* and ∆*dpr* strains, 5 mL of refreshed GAS cells at the mid-logarithmic phase was collected and washed in M200 medium. For the GAS hydrogen peroxide (H_2_O_2_) challenge *in vitro*, the GAS pellets were collected from a 5 mL culture after the H_2_O_2_ challenge and washed in M200 medium. The washed GAS pellets were immediately preserved in the RNA*later* stabilization solution (Thermo Fisher Scientific) and stored at −80°C until use. The RNA*later* solution of preserved GAS pellets was discarded, and the pellets were resuspended in the lysozyme buffer (Geneaid, New Taipei City, Taiwan) containing sodium hypochlorite-treated silica beads (0.1 mm) (Sigma, St. Louis, USA). Subsequently, the homogeneous supernatant of mechanically vortex-broken cells was applied to the RNA isolation column of GENEzol TriRNA Bacterial Kit (Geneaid), according to the manufacturer’s instructions.

For dual RNA-seq analyses, HMEC-1 cells containing intracellular GAS were collected from 6-well plates at 1 h post-infection, followed by trypsin-EDTA (Thermo Fisher Scientific) detachment and washing in M200 medium. The cell pellets were preserved in the RNA*later* stabilization solution at −80°C until use. The RNA isolation was carried out as described above.

The quality and quantity of the total RNA sample were validated using RNA agarose gel electrophoresis and NanoDrop ND-1000 absorbance measurement (OD_260/280_ and OD_260/230_) (Thermo Fisher Scientific), respectively. Furthermore, the total RNAs for dual RNA-seq analysis were evaluated using the Eukaryote Total RNA Nano Kit for the 2100 Bioanalyzer system (Agilent, Santa Clara, USA). The RNAs that obtained the RNA integrity number greater than 9 were used for subsequent library preparation of dual RNA-seq analysis.

### Reverse transcription and cDNA-qPCR analysis

The total RNA (0.5 µg) was converted to cDNA using the ReverTra Ace qPCR RT Kit (TOYOBO, Osaka, Japan). The resultant cDNA was diluted 20-fold in elution buffer (50 mM Tris-HCl, pH 8.0) and verified to have no gDNA contamination. The cDNA samples were stored at −20°C until use. Two micrograms of diluted cDNA was applied for qPCR analysis according to the SYBR green master mix protocol of the Applied Biosystems (Thermo Fisher Scientific). We performed and analyzed the qPCR assay using the StepOnePlus platform. Relative gene expression (fold change) was calculated using the formula 2^-∆∆Ct^ when compared to *gyrA* as the reference internal control.

### Dual RNA-seq analysis

High-quality total RNAs (HMEC-1 cells and GAS) were co-isolated from the infected-HMEC-1 cells containing intracellular GAS cultured on 6-well plates at 1 h post-infection. We collected the RNA samples from HMEC-1 cells, GAS WT-infected HMEC-1 cells, and GAS ∆*perR*-infected HMEC-1 cells from four independent infection experiments. The total RNAs were treated with DNase I and ribosomal RNA depletion (Ribo-Zero rRNA Removal Kits; Illumina, USA), prior to library preparation (Stranded Total RNA Prep kit; Illumina) and NextSeq2000 sequencing (2 × 100 bp paired-end sequencing) (Illumina). The raw fastq data sets were deposited to the NCBI Gene Expression Omnibus (GEO) repository (http://www.ncbi.nlm.nih.gov/geo/). We obtained ~80.4 million read pairs for each library. Raw sequencing data quality was analyzed using Fastp (version 0.23.2), and the sequencing adapters and low-quality reads were trimmed and removed using STAR (version 2.7.9a) (33). The reads were aligned to the GAS NZ131 reference genome (NCBI accession number, NC_011375.1) and human reference genome (hg38) using the featureCounts (version 2.0.4). The differential gene expression analysis was performed separately for HMEC-1 cells and GAS and normalized using DESeq2. The GAS genes with a *P*_adj_ < 0.05 and a log_2_ fold change ≥ 1 or ≤ −1 were considered to be differentially expressed genes (DEGs). Gene ontology (GO) term enrichment analysis of the DEGs was performed using the Blast2GO tool (34).

### PerR protein purification

The PerR expression clone (pET21b-perR-exp-FR2) was generated by ligation of an *Nde*I/*Xho*I-cleaved *perR* amplicon (468 bp; primers: perR-exp-F-*Nde*I and perR-exp-R2-*Xho*I) (Table S1) with *Nde*I/*Xho*I-cleaved pET-21b. The Sanger sequencing verified pET21b-perR-exp-FR2 was then transformed into *Escherichia coli* BL21 (DE3) strain for overexpression and purification. To express the 6×His-tagged PerR protein, IPTG was added (final 0.5 mM) to the culture at an optical density (OD_600_) of 0.6, followed by incubation at 25°C for 4 h. The cells were harvested by centrifugation at 10,000 *g* for 20 min and resuspended in buffer A (20 mM HEPES and 200 mM NaCl, pH 7.5). Cell lysis was done on ice by sonication (ChromTech, UP-500; Apple Valley, MN, USA). The supernatant was applied to a Ni-NTA affinity column (Ni Sepharose 6 Fast Flow, Cytiva, Marlborough, MA, USA), and non-specific proteins were removed by washing with buffer A (60 mM imidazole). The PerR protein was eluted using buffer A (300 mM imidazole). The PerR-eluted fractions were pooled and buffer-exchanged into buffer B (20 mM Tris-HCl, 150 mM NaCl, 0.5 mM EDTA, 5% glycerol, and 3 mM DTT, pH 7.5) using Amicon Ultra centrifugal filters (10 kDa MWCO, Millipore, Burlington, MA, USA). The purified PerR protein was stored at 4°C until use.

### Electrophoretic mobility shift assay

The interaction between PerR and promoter DNA regions, which either contain or lack the putative Per box, was analyzed using electrophoretic mobility shift assay (EMSA) as previously described (35, 36). Briefly, promoter DNA regions were PCR-amplified with various primer sets (Table S1) using the NZ131 WT genome as the template. The length of amplicons ranges from 80 to 130 bp. Promoters 1 and 2 are the *pmtA* regions. Promoter 1 (primers: pmtA_pro_EMSA_F1 and R1) was amplified to obtain Per box, while promoter 2 (primers: pmtA_pro_EMSA_F3 and R3) contains no Per box. Promoters 3 and 4 are *dpr* regions. Promoter 3 (dpr_pro_EMSA_F3 and R3) has Per box, while promoter 4 (primers: dpr_pro_EMSA_F2 and R2) has no Per box. The *adcA* promoter 5 (primers: adcA_pro_EMSA_F1 and R1) has no Per box but obtains AdcR motif; promoter 6 (adcA_pro_EMSA_F1 and R2) has no Per box or AdcR motif.

In general, purified PerR protein and DNA promoters were mixed in a 20 µL reaction containing binding buffer (20 mM Tris, pH 8.0; 50 mM KCl; 10 μM ZnCl_₂_; 5% glycerol; and 50 µg/mL bovine serum albumin) and incubated at 4°C for 30 min. Reaction mixtures were then resolved on 6% native polyacrylamide gels in Tris-borate buffer (45 mM Tris-base and 45 mM boric acid, pH 8.3). Gels were stained with DNA-safe staining dye (DNA View; Tools, New Taipei City, Taiwan) and visualized using a UV imaging system.

### Intracellular metal ion measurement

Overnight grown GAS strains were refreshed in TSBY broth to an OD_600_ of 0.9–1.2 (early stationary phase). The bacterial pellets (2 OD_600_) were collected and washed twice in PBS buffer. After removing the supernatant, the pellets were digested in 50 µL of 14 M nitric acid (HNO_3_) at RT for 18 h, followed by adding 50 µL sterile deionized water. The samples were analyzed for iron and zinc by inductively coupled plasma mass spectrometry (ICP-MS) analysis at the Instrumentation Center at National Tsing Hua University. Total parts per billion values for metal were converted to nanomolar in samples.

### Flow cytometric analysis

For cellular total ROS measurement, HMEC-1 cells were collected from 6-well plates after 1 h of infection, followed by trypsin-EDTA detachment and washing in PBS buffer. The pelleted cells were resuspended in the staining buffer (2% FBS/0.1% NaN_3_ in PBS buffer) containing 5 µM ROS dye, carboxymethyl-H_2_-dichlorofluorescein diacetate (CM-H_2_DCFDA) (Thermo Fisher Scientific) for 10 min at RT in the dark. After centrifugation (500 *g* for 4 min at 4°C), the stained cells underwent fixation in 4% paraformaldehyde for 10 min at RT and ultimately were resuspended in the staining buffer for flow cytometric detection (CytoFLEX, Beckman). The signal of CM-H_2_DCFDA dye was collected with excitation wavelength set at 488 nm and the emission set at FITC channel. The mean fluorescence intensity (MFI) of ROS detection was analyzed using CytExpert software (Beckman).

### Western blotting analysis

HMEC-1 cells were seeded in a 10 cm dish overnight and infected with GAS WT or ∆*perR* mutant for 1 h, according to the GAS infection model described above. The GAS-infected cells were trypsin-EDTA detached and immediately preserved in RNA*later* stabilization solution at −80°C until use. In brief, the GAS-infected HMEC-1 cells were resuspended in 100 µL of chilled Buffer A (200 mM NaCl and 20 mM Tris-HCl, pH 7.5) and transferred to a 2 mL microtube (SSIBio#2330-00, Scientific Specialties, USA) containing sterile 100–150 mg zirconia/silica beads (0.1 mm diameter, cat# 11079101z, BioSpec Products, USA) for homogenization. We obtained homogeneous lysates using a bead beater (model Precellys 24 Touch, Bertin Instruments, France) with the setting (2 cycles of 30 s of beading at 5,500 rpm and 3 min of chilling on ice). After centrifugation (12,000 *g* for 10 min at 4°C), the lysate (supernatant) was collected for Bradford protein assay (Bio-Rad, USA), Coomassie blue staining, and Western blotting. The transferred proteins on the polyvinylidene fluoride membrane were immersed in blocking buffer (5% milk) for 1 h, followed by incubation at 4°C overnight with primary anti-GAS Dpr mouse polyclonal antibody (19) and anti-β actin (66009-1-Ig, mouse; Proteintech, USA). The next day, the membranes were washed several times and incubated with HRP-conjugated secondary antibodies at RT for 1 h. The blots were visualized using Western Lightnin Plus Chemiluminescence Reagent (PerkinElmer, Waltham, MA, USA).

### Agar spot assay

For agar spot analysis, GAS strains were refreshed in TSBY broth with appropriate antibiotics to reach the mid-logarithmic phase, then diluted in TSBY broth to obtain 10^6^–10^1^ CFU per 5 µL of culture. We deposited 5 µL of GAS culture with various CFU concentrations onto TSBY agar plates, supplemented with ZnSO_4_ (0.25, 0.5, 1, and 2 mM) or zinc chelator TPEN (25 and 50 µM). CFU observation was performed after 20 h of incubation at 37°C in 5% CO_2_.

### Data visualization and statistical analysis

The growth curves of GAS, the volcano plot of the DEGs, and the bar plot of GO term enrichment analysis were visualized using the R package (version 4.3.2) and ggplot2 (version 3.4.4). Graphs of cDNA-qPCR analysis were generated using GraphPad Prism (version 8). Statistical analysis was performed using an unpaired Student’s *t* test, multiple *t* test, one-way or two-way analysis of variance (ANOVA) (GraphPad Prism). Statistical significance was represented as *P < 0.05; **P < 0.01; ***P < 0.001; and ****P < 0.0001.

The biological assembly of Dpr was obtained from the Protein Data Bank (https://www.rcsb.org/) (37–39) and visualized using PyMOL (version 3.1.2). Electrostatic surface potentials were calculated using the APBS electrostatics (PyMOL). Structural comparisons were performed to examine Zn^2+^-bound, Fe^2+^-bound, and metal-free states of monomeric Dpr protein.

## RESULTS

### Disruption of GAS *perR* enhances H_2_O_2_ tolerance but diminishes GAS survival in HMEC-1 cells

**Fig 1:**
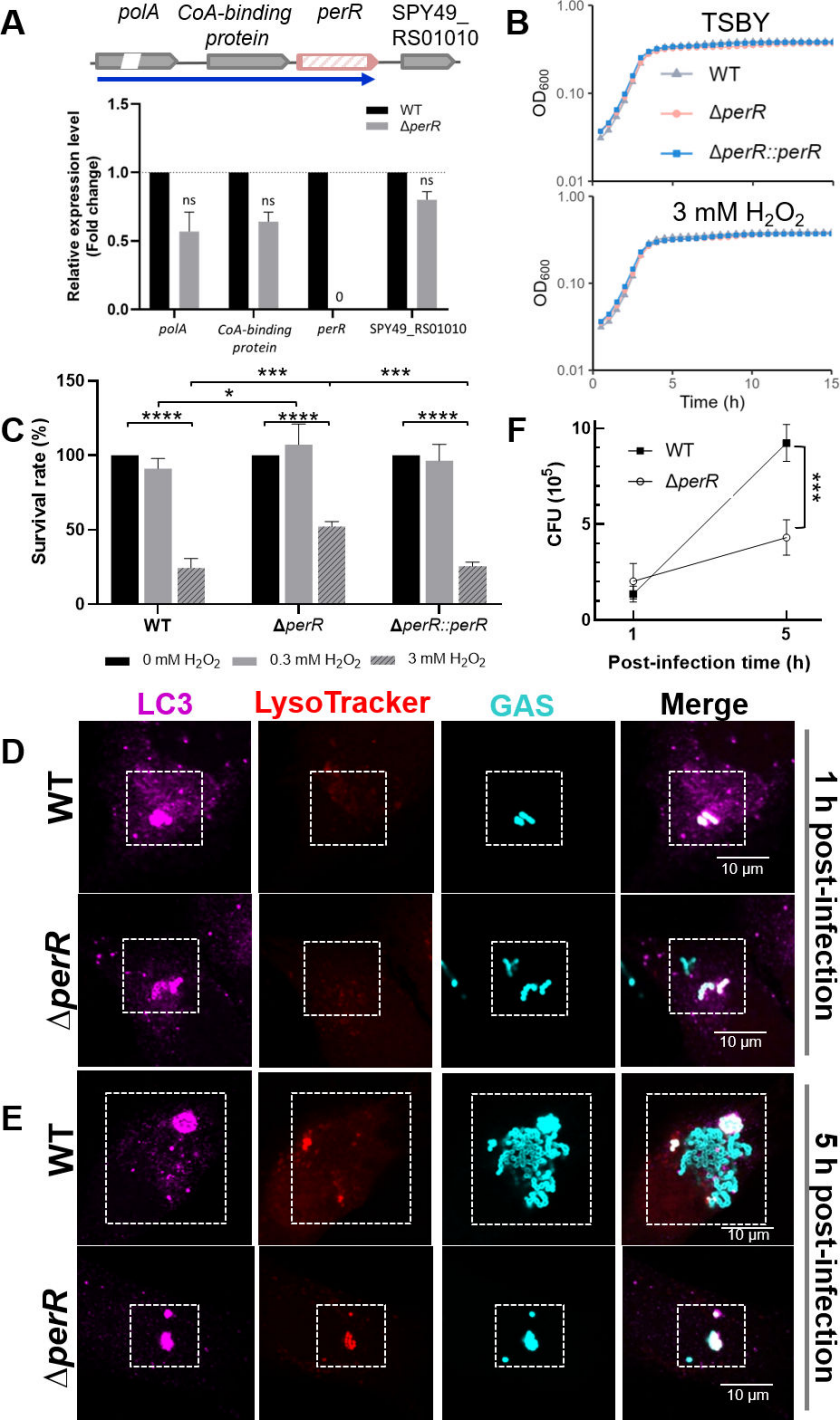
Impact of *perR* disruption on GAS pathogenesis. (**A**) Polarity assessment of GAS Δ*perR* mutant. The mRNA expression of *perR* and its upstream and downstream genes was validated in the GAS NZ131 WT and Δ*perR* mutant using cDNA-qPCR analysis. In the Δ*perR* mutant, the relative mRNA expression levels (fold changes) of *polA,* the gene coding for CoA-binding protein, and the SPY49_RS01010 gene were insignificant and comparable to the WT strain. This indicates a successful in-frame deletion of the Δ*perR* mutant, which possesses no polar effect. (**B**) Growth of GAS strains in TSBY medium supplemented with hydrogen peroxide. The GAS WT, Δ*perR*, and Δ*perR::perR* complemented strains were inoculated in TSBY and TSBY with 3 mM H_2_O_2_ media at 37°C for at least 15 h under ambient atmosphere. The optical density (OD_600nm_) was recorded every 30 min. (**C**) GAS survival after H_2_O_2_ challenge. GAS strains were refreshed to the mid-logarithmic phase and exposed to M200 medium supplemented with H_2_O_2_ (0.3 and 3 mM) as peroxide challenge. The survival rate of GAS strains was converted from the ratio of the viable CFU counts. Statistical significance was assessed using two-way ANOVA with Tukey’s multiple comparisons test (*P < 0.05; ***P < 0.001; and ****P < 0.0001). All data are presented as mean values ± SD of three independent biological repeats. (**D and E**) Clearance of GAS from the infected HMEC-1 cells. HMEC-1 cells were infected with the GAS WT or Δ*perR* strain. Immunofluorescence microscopic analysis was performed to determine GAS clearance via the LC3-positive GAS-containing vacuoles in HMEC-1 cells. The microscopic images were captured after HMEC-1 cells were infected with the WT or Δ*perR* mutant at (**D**) 1 and (**E**) 5 h post-infection. Anti-LC3 (magenta) was used to determine the location of LC3^+^ phagosomes. LysoTracker (red) was an acidotropic indicator, and anti-GAS (cyan) bound to the carbohydrates of GAS. (**F**) Multiplication of GAS during cell invasions. The intracellular viable bacteria were cultivated and enumerated by CFU-based assay at 1 and 5 h post-infection. Statistical significance was assessed using two-way ANOVA with Sidak’s multiple comparisons test (***P < 0.001). Data (**D and E**) are representative of three independent biological repeats. Data (**F**) are presented as mean values ± SD of three independent biological repeats.

We first constructed GAS NZ131 Δ*perR* in-frame deletion mutant, which was validated using whole-genome sequencing analysis and cDNA-qPCR analysis. Neither spurious mutation nor polarity was detected in the Δ*perR* mutant (accession: PRJNA1357628) (Fig. 1A) (Table S2). Next, we examined the growth of the GAS strains inoculated in TSBY and 3 mM H_2_O_2_ *in vitro* (Fig. 1B). The similar growth rates of the WT, the Δ*perR* mutant, and an in *trans*-complemented Δ*perR::perR* under these conditions implied that *perR* deletion does not significantly affect GAS growth in the media tested. Moreover, in the 3 mM H_2_O_2_ challenge for 30 min, we found a higher survival rate of the Δ*perR* mutant, in comparison with the WT and complemented mutant (Fig. 1C). Further cDNA-qPCR analysis of the three strains revealed that PerR modulates H_2_O_2_-induced ROS stress, at least in part through PerR’s suppression of *dpr*, *ahpC*, *ahpF*, and *sodA* (Fig. S1). We wondered whether the Δ*perR* mutant could survive in HMEC-1 cells, where GAS WT induces cellular ROS-triggered LAP as reported previously (13). To answer this question, we performed immunofluorescence microscopic analysis to detect LC3-positive GAS-containing vacuoles in HMEC-1 cells. The HMEC-1 cells were infected by the WT and Δ*perR* mutant for 1 h (Fig. 1D) and 5 h (Fig. 1E). When the signals of anti-GAS, anti-LC3, and LysoTracker (acidic pH indicator) were co-localized, the co-localization showed the presence of acidified GAS-containing vacuoles during the host clearance process. In the WT-infected HMEC-1 cells, we observed low acidification of the LC3^+^ GAS-containing vacuoles at both time points (Fig. 1D and E). A 7.2-fold CFU increase of GAS WT was observed between these 1- and 5-h points (Fig. 1F). Interestingly, in the Δ*perR*-infected HMEC-1 cells, the sufficient acidification of LC3^+^Δ*perR*-containing vacuoles was detected 5 h after infection (Fig. 1E), with a mere 2.4-fold multiplication of CFU when compared to that of 1 h post-infection (Fig. 1F). Nonetheless, the cytotoxicity of GAS-infected HMEC-1 cells was low (less than 5%) (Fig. S2). These results indicate that HMEC-1 cells constrain the growth of the Δ*perR* strain, and this discovery led us to investigate the molecular mechanism of host cell defenses and GAS counteraction during invasion.

### Transcriptome profile of the Δ*perR* mutant during invasion

**Fig 2:**
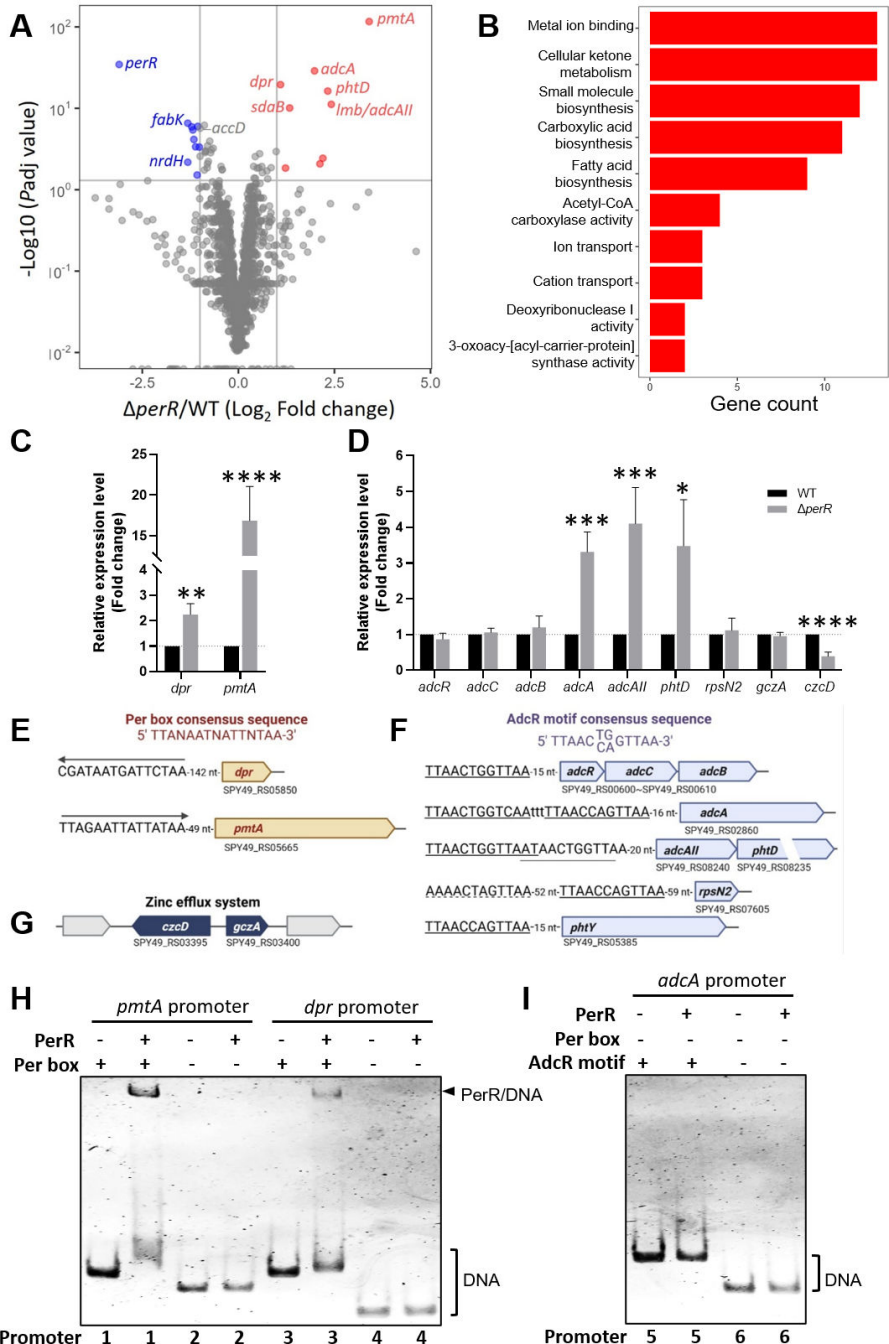
GAS transcriptome analysis and promoter examination. HMEC-1 cells were infected with the WT or Δ*perR* mutant at 1 h post-infection, followed by RNA isolation and dual RNA-seq analysis. (**A**) The volcano plot analysis. The DEGs in the Δ*perR* mutant are indicated as upregulated (red) and downregulated (blue) genes. Statistically significant DEGs (log_2_ fold change ≥ 1 or ≤ −1 with *P*_adj_ ≤ 0.05) are labeled. (**B**) GO term enrichment analysis. The GO analysis shows the selective GO terms of biological processes. (**C**) The cDNA-qPCR validation on the *dpr* and *pmtA* expression in the GAS WT and Δ*perR* mutant recovered from the infected HMEC-1 cells at 1 h post-infection. (**D**) The cDNA-qPCR validation on the expression of zinc acquisition and zinc efflux genes in the GAS WT and Δ*perR* mutant recovered from the infected HMEC-1 cells at 1 h post-infection. Statistical significance was assessed using two-way ANOVA with Sidak’s multiple comparisons test (*P < 0.05; **P < 0.01; ***P < 0.001; and ****P < 0.0001). All data are presented as mean values ± SD of three independent biological repeats. (**E**) Promoter analysis of PerR-mediated *dpr* and *pmtA* genes. A Per box operator is identified in the promoter regions of the *dpr* and *pmtA* genes with different orientations. (**F**) Promoter analysis of GAS zinc acquisition system. AdcR is a repressor for zinc acquisition and adhesion competence. The presence of one AdcR motif is found in the zinc acquisition system (*adcRCB* operon, *phtY*, and *rpsN2* genes). There are two AdcR motifs found in the zinc acquisition system, *adcA* and *adcAII-phtD* genes, which are also part of PerR-mediated zinc homeostasis genes. (**G**) Promoter analysis of GAS zinc efflux systems. The zinc efflux system contains *czcD* coding for the zinc exporter and *gczA* coding for the *czcD* activator. (**H and I**) The binding of PerR with promoter regions using the EMSA. (**H**) PerR-*pmtA*/*dpr* promoter binding. The promoters of *pmtA* and *dpr* were amplified to contain Per box (promoters 1 and 3) and no Per box (promoters 2 and 4). When PerR was added to interact with the promoters, PerR-DNA mobility shifts were observed (promoters 1 and 3) but not in promoters 2 and 4 with no Per box. (**I**) PerR-*adcA* promoter binding. The *adcA* promoter contains the AdcR motif (promoter 5) but no Per box (promoters 5 and 6). When PerR was added, there was no mobility shift of PerR-*adcA* promoter binding.

To discover how the growth of GAS Δ*perR* was constrained by HMEC-1 cells, we performed a dual RNA-seq analysis. As shown in Fig. 2A and Table S3, we detected 22 DEGs (fold change ≥ 2 or ≤ 0.5 with *P*_adj_ ≤ 0.05) in the Δ*perR* mutant recovered from the infected HMEC-1 cells. In brief, there were 10 upregulated and 12 downregulated DEGs (Fig. 2A; Table S3). The subsequent GO term enrichment analysis (Fig. 2B) revealed that the majority of the upregulated genes were associated with metal ion binding, whereas the downregulated genes were mostly involved in carboxylic acid biosynthesis. We conducted cDNA-qPCR analysis to validate our RNA-seq findings. We found that the correlation of relative expressions in the selective genes obtained from the dual RNA-seq and cDNA-qPCR analyses showed reliability with an *R*^2^ value of 0.88 (Fig. S3). The qPCR analysis revealed that at least five GAS genes (*dpr*, *pmtA*, *adcA*, *lmb/adcAII*, and *phtD*) (Table S3; Fig. 2C and D) were significantly upregulated in the Δ*perR* mutant compared to the WT strain in the HMEC-1 infection model. However, the expression of downregulated GAS genes that encode glutaredoxin-like proteins and carboxylic acid biosynthesis did not decrease significantly, but zinc efflux *czcD* was downregulated in the Δ*perR* mutant (Table S3).

In the cell-recovered Δ*perR* mutant, the increased *dpr* and *pmtA* were validated (Fig. 2C), suggesting that they were involved in PerR-regulated iron-related ROS stress and iron homeostasis. The *dpr* gene encodes a Dps-like peroxide resistance protein with an iron and zinc-binding ferritin-like antioxidant (19, 20). The *pmtA* gene encodes a heavy metal translocating P_1B-4_-type ATPase for iron efflux but not zinc efflux (22, 23). We then detected a putative Per box sequence of PerR binding with reverse orientation in the *dpr* promoter, while a putative canonical Per box was found in the promoter of the *pmtA* gene (Fig. 2E). In addition to iron-related oxidative stress, we identified certain genes of the zinc acquisition system and zinc efflux (Fig. 2D, F, and G) (25–30). The enhanced genes have two AdcR motifs with no Per box in the promoters of *adcA*, *lmb/adcAII*, and *phtD* that encode the zinc ABC transporter substrate-binding protein, the laminin-binding protein/zinc ABC transporter substrate-binding protein II, and the pneumococcal-type histidine triad protein D, respectively. We then performed the EMSA to examine whether PerR binds to the promoter of the *pmtA*, *dpr*, or *adcA*. As shown in Fig. 2H, PerR made band shifts due to PerR-DNA binding, which only occurred when the promoter regions contained Per box (see *pmtA* promoter 1 and *dpr* promoter 3). Meanwhile, no DNA shift was observed in the PerR-*adcA* promoters 5 and 6 (Fig. 2I), indicating that the *adcA* expression is not directly controlled by PerR. Together, our findings show that there is a possibility that the PerR regulon is associated with iron and zinc-related ROS stress through *pmtA* and *dpr* overexpression, along with metal ion (iron and zinc) homeostasis for GAS fitness during cell invasions.

### GAS Δ*perR* confronts iron depletion, ROS stress, intracellular Dpr overexpression, and iron/zinc adaptation

**Fig 3:**
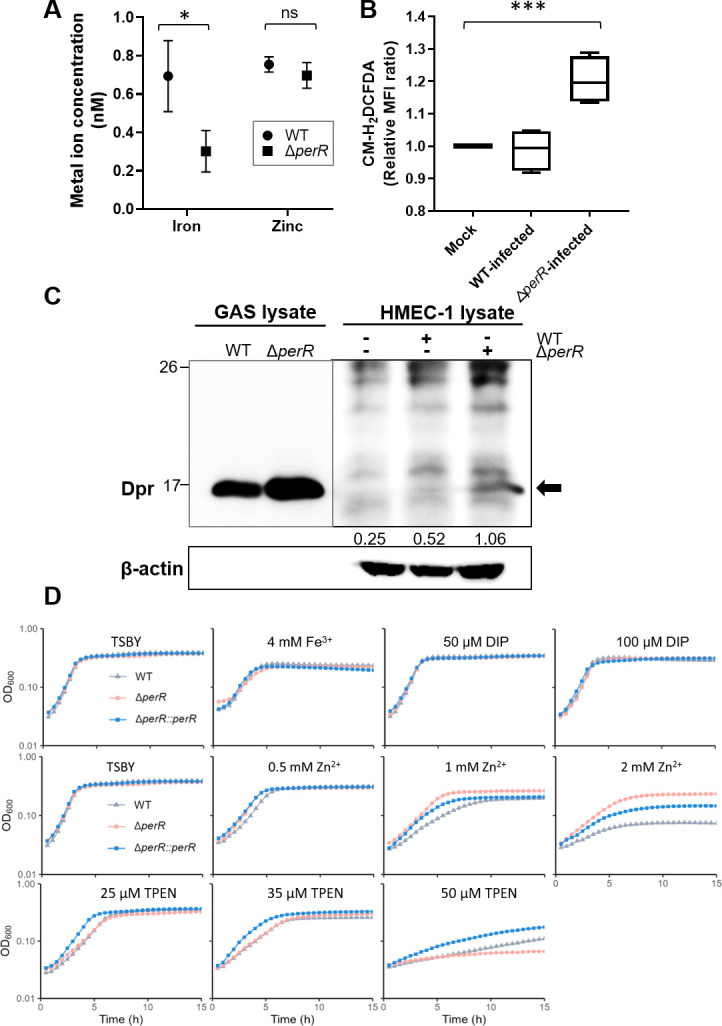
GAS confronts iron depletion, ROS stress, Dpr overexpression, and iron/zinc intoxication and depletion. (**A**) Metal ion determination by ICP-MS analysis. GAS WT (spheres) and Δ*perR* (squares) were grown in TSBY medium, and the intracellular contents of iron and zinc were measured. Statistical significance was assessed using a two-sample *t*-test (*P < 0.05). All data are presented as mean values ± SD of three independent biological repeats. (**B**) Generation of cellular ROS in the GAS-infected HMEC-1 cells. HMEC-1 cells were collected at 1 h after being infected with GAS WT or Δ*perR* with an MOI of 50, followed by CM-H_2_DCFDA staining for cellular ROS signal. The signal of CM-H_2_DCFDA was detected using flow cytometry and calculated as MFI. Statistical significance was assessed using a two-sample *t*-test (***P < 0.001). The data shown are the relative mean values ± SD of four independent biological experiments. (**C**) Western blot analysis of GAS Dpr proteins. GAS WT- or Δ*perR*-infected HMEC-1 cells were collected at 1 h post-infection, followed by homogenization to extract total proteins, which contained the lysate of HMEC-1 cells and GAS. We examined the expression of GAS Dpr proteins (arrow) and HMEC-1 β-actin (control). The relative expression of Dpr proteins versus β-actin was calculated using ImageJ software. The data are representative of three independent experiments. (**D**) Growth of GAS WT (gray triangles), Δ*perR* mutant (red spheres), and Δ*perR::perR* mutant (blue squares) in TSBY broth supplemented with FeCl_3_ (4 mM), iron chelator 2,2′-dipyridyl (DIP) (50 and 100 μM), ZnSO_4_ (0.5, 1, and 2 mM), or zinc chelator TPEN (25, 35, and 50 μM). The GAS strains were refreshed and inoculated in TSBY broth with various supplements for at least 15 h at 37°C.

To understand whether the deletion of *perR* affects the iron and zinc contents inside GAS, we measured the intracellular iron and zinc of the GAS WT and Δ*perR* mutant using ICP-MS analysis. When grown in TSBY medium, the intracellular iron content of the Δ*perR* mutant was significantly lower than that of the WT strain (*P* < 0.05), but the intracellular zinc content remained unchanged (*P* > 0.05) (Fig. 3A). This result suggests that the Δ*perR* mutant upregulates *pmtA* and thus causes decreased iron content in the cytosol. With this in mind, we determined whether the Δ*perR*-infected HMEC-1 cells encounter GAS-induced cellular ROS. We performed flow cytometric analysis using a ROS dye, CM-H_2_DCFDA. Figure 3B shows that cellular ROS production increased slightly in the Δ*perR*-infected HMEC-1 cells, indicating that the GAS Δ*perR* mutant confronted cellular ROS stress inside endothelial cells. Additionally, our Western blot analysis revealed a higher Dpr expression in the Δ*perR*-infected HMEC-1 cells (1.06-fold) than in the WT-infected cells (0.52-fold) at 1 h post-infection (Fig. 3C). This demonstrated that Dpr may aid in GAS’s survival of ROS stress, as antioxidant Dpr acts like iron and zinc-binding ferritin protein (19, 20).

We next examined whether GAS PerR is involved in iron/zinc adaptation and how the Δ*perR* mutant develops during iron/zinc overload and depletion. We performed *in vitro* bacterial growth assays by supplementing TSBY with ferric chloride (FeCl_3_), 2,2′-dipyridyl (DIP; a Fe^2+^ chelator), zinc sulfate (ZnSO_4_), or zinc chelator TPEN. We used a broth growth curve assay (Fig. 3D) and an agar spot assay (Fig. S4). When TSBY broth was added with FeCl_3_ (0.5–4 mM) or DIP (10–100 μM), similar growth patterns were observed among the WT, Δ*perR* mutant, and Δ*perR:perR* mutant (Fig. 3D; Fig. S4). With an increase of zinc, only the Δ*perR* mutant grew better than the others under a higher zinc concentration (1 and 2 mM) (Fig. 3D; Fig. S4). However, all the strains that we tested were susceptible to zinc deprivation when TPEN (50 μM) was applied (Fig. S4). Strikingly, the growth curve of the Δ*perR* mutant appeared to be severely inhibited when the zinc in the media was chelated by 50 μM TPEN (Fig. 3D). From these findings, we suggest that the deletion of PerR in the GAS makes the mutant strain more adaptable to zinc intoxication but more susceptible to zinc depletion with a trace change of zinc availability, presumably that zinc adaptation is associated with the Dpr overexpression in the Δ*perR* mutant.

**Fig 4:**
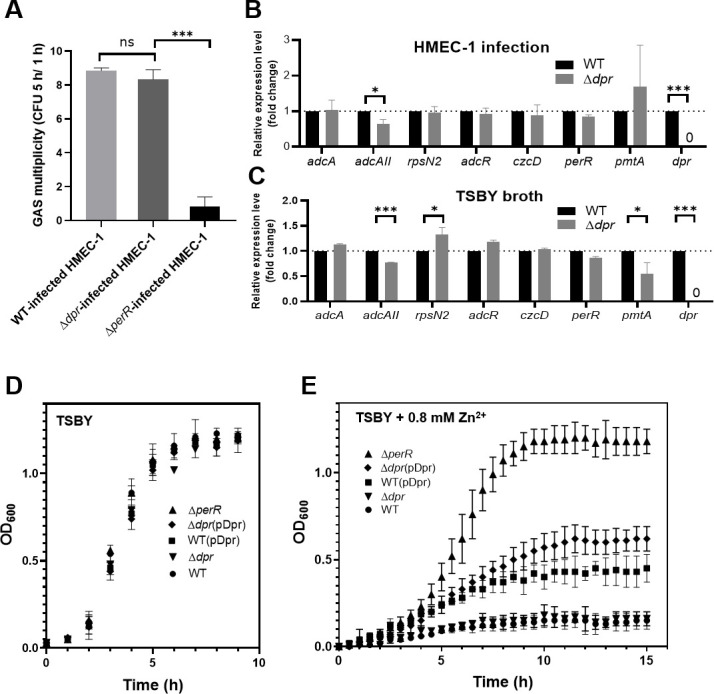
Phenotypical characterization of the Δ*dpr* mutant. (**A**) Survival of the WT, Δ*dpr* mutant, and Δ*perR* mutant during HMEC-1 cell invasions. The intracellular viable bacteria were determined using a CFU-based assay at 1 and 5 h post-infection. Statistical significance was assessed using a multiple *t*-test (***P < 0.001). (**B and C**) The cDNA-qPCR analysis on the gene expression of zinc acquisition, zinc efflux, and PerR regulon in the GAS WT and Δ*dpr* strains. GAS strains were recovered from (**B**) HMEC-1 cells at 1 h post-infection and from (**C**) TSBY cultivation until the mid-logarithmic phase. Data (**B and C**) are presented as mean values ± SD of at least two independent biological repeats. Statistical significance was assessed using a multiple *t*-test (*P < 0.05; ***P < 0.001). (**D and E**) Growth of GAS WT (sphere), WT(pDpr) (Dpr expression, square), Δ*perR* (Dpr overexpression, triangle), Δ*dpr* (inverted triangle), and Δ*dpr*(pDpr) (Dpr expression, diamond) strains in (**D**) TSBY broth or (**E**) TSBY broth supplemented with 0.8 mM ZnSO_4_. The GAS strains were refreshed and inoculated for at least 9 h at 37°C. Data shown are representative of at least three independent biological experiments.

### Expression of Dpr facilitates GAS’s zinc storage

To examine our hypothesis that Dpr is involved with zinc adaptation, we analyzed the Dpr protein sequence alignment of the strains NZ131 and A20. The alignment showed 100% identical sequences (Fig. S5). Furthermore, the Dpr protein structure is shown in Fig. S6A. It is a hollow spherical dodecameric complex of the assembled Dpr, like a repository, which is a cavity where iron and zinc ions are stored (37). We also found a conformational similarity between Zn^2+^-bound, Fe^2+^-bound, and metal-free monomeric Dpr structures (Fig. S6B) (37, 38). These results implied that the Dpr of NZ131 is capable of iron and zinc binding like A20 Dpr. In addition to the Dpr structural analysis, we generated an isogenic Δ*dpr* mutant without polarity (Fig. S7) to test its involvement with zinc. In the HMEC-1 cell infection model, the Δ*dpr* mutant maintained a similar multiplicity compared to that of the WT strain (Fig. 4A). The expression of the zinc acquisition and zinc efflux genes of the Δ*dpr* mutant, when grown either inside HMEC-1 cells or in TSBY broth, was mostly unchanged compared to the WT strain (Fig. 4B and C). Interestingly, the downregulated *adcAII* in both conditions and upregulated *rpsN2* in the TSBY condition were observed in the Δ*dpr* mutant. It is noticeable that *pmtA* expression was reduced in TSBY broth but increased inside the HMEC-1 cells, implying that PmtA helps the Δ*dpr* mutant confront ROS stress during infection.

We attempted to understand whether or not Dpr aids in GAS fitness during zinc adaptation. We generated overexpressed Dpr by introducing pDpr (Dpr-expressing plasmid) into the WT and Δ*dpr* strains, respectively, to form the WT(pDpr) strain and the Δ*dpr*(pDpr) complemented mutant. In comparison with the WT, Δ*dpr* mutant, and Δ*perR* mutant, when inoculated in TSBY broth, these five strains tested grew well, displaying a similar growth rate (Fig. 4D). Contrarily, when supplemented with 0.8 mM ZnSO_4_ in TSBY broth, it was clearly shown that the WT and Δ*dpr* mutant strains grew poorly, but not the Δ*perR* mutant (Dpr overexpression), the WT(pDpr) strain, or the Δ*dpr*(pDpr) complemented mutant (Fig. 4E). Therefore, our results suggest that Dpr can bind zinc and play a role as a repository for zinc to facilitate GAS adaptation in zinc intoxication.

### GAS encounters zinc deprivation inside the vacuoles of endothelial cells

**Fig 5:**
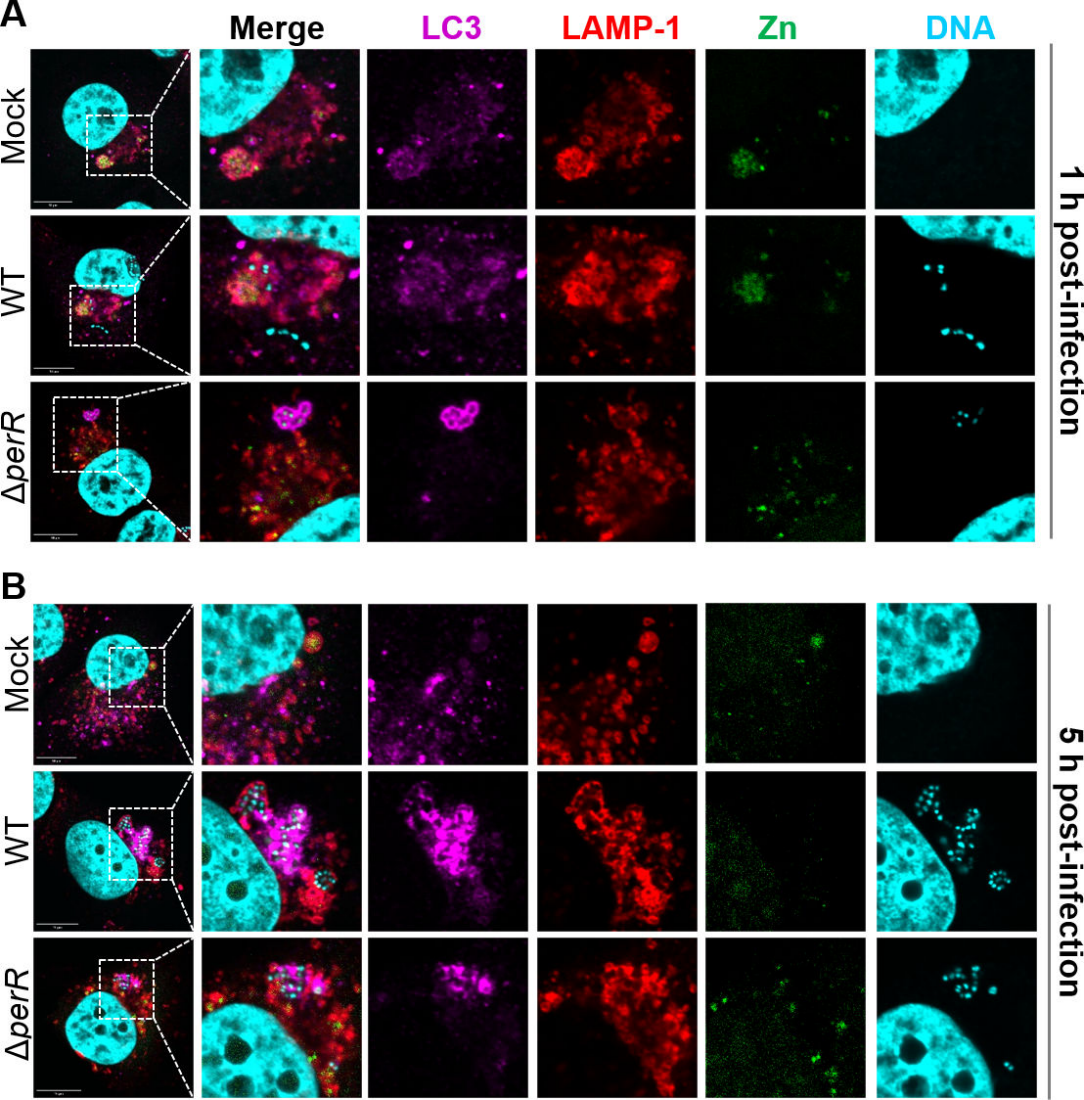
Distribution of cellular zinc in the GAS-containing vacuoles of endothelial cells. Immunofluorescence microscopic analysis is conducted to detect zinc distribution when GAS WT or Δ*perR* mutant was enclosed in the LC3^+^LAMP-1^+^ vacuoles of HMEC-1 cells. The microscopic images were captured after HMEC-1 cells were infected with the WT or Δ*perR* mutant at (**A**) 1 and (**B**) 5 h post-infection. Anti-LC3 (magenta) is used to determine the location of LC3^+^ phagosomes. LAMP-1 (red) is a lysosomal indicator, free zinc (green) is detected using zinc FluoZin-3 AM, and nucleic acid (cyan) is stained using DAPI. Scale bars, 10 µm.

To further understand whether the response of endothelial cells to GAS infection is associated with zinc, we investigated cellular zinc using immunofluorescence microscopic analysis. The co-localized LC3^+^LAMP-1^+^ vacuoles show fused LAPosomes and lysosomes. One hour after infection, the LC3^+^LAMP-1^+^ GAS WT-containing vacuoles showed a trace signal of zinc ions (Fig. 5A). In a similar phenomenon, there was no intense zinc signal in the LC3^+^LAMP-1^+^ GAS Δ*perR*-containing vacuoles. A similar zinc deprivation was observed in the LC3^+^LAMP-1^+^ GAS-containing vacuoles under GAS WT- and Δ*perR*-infected HMEC-1 cells at the 5 h post-infection point (Fig. 5B). These results demonstrate that the WT and Δ*perR* mutant encountered zinc limitation inside the fused phagolysosomes that possibly made the Δ*perR* mutant more vulnerable to zinc restriction.

## DISCUSSION

**Fig 6:**
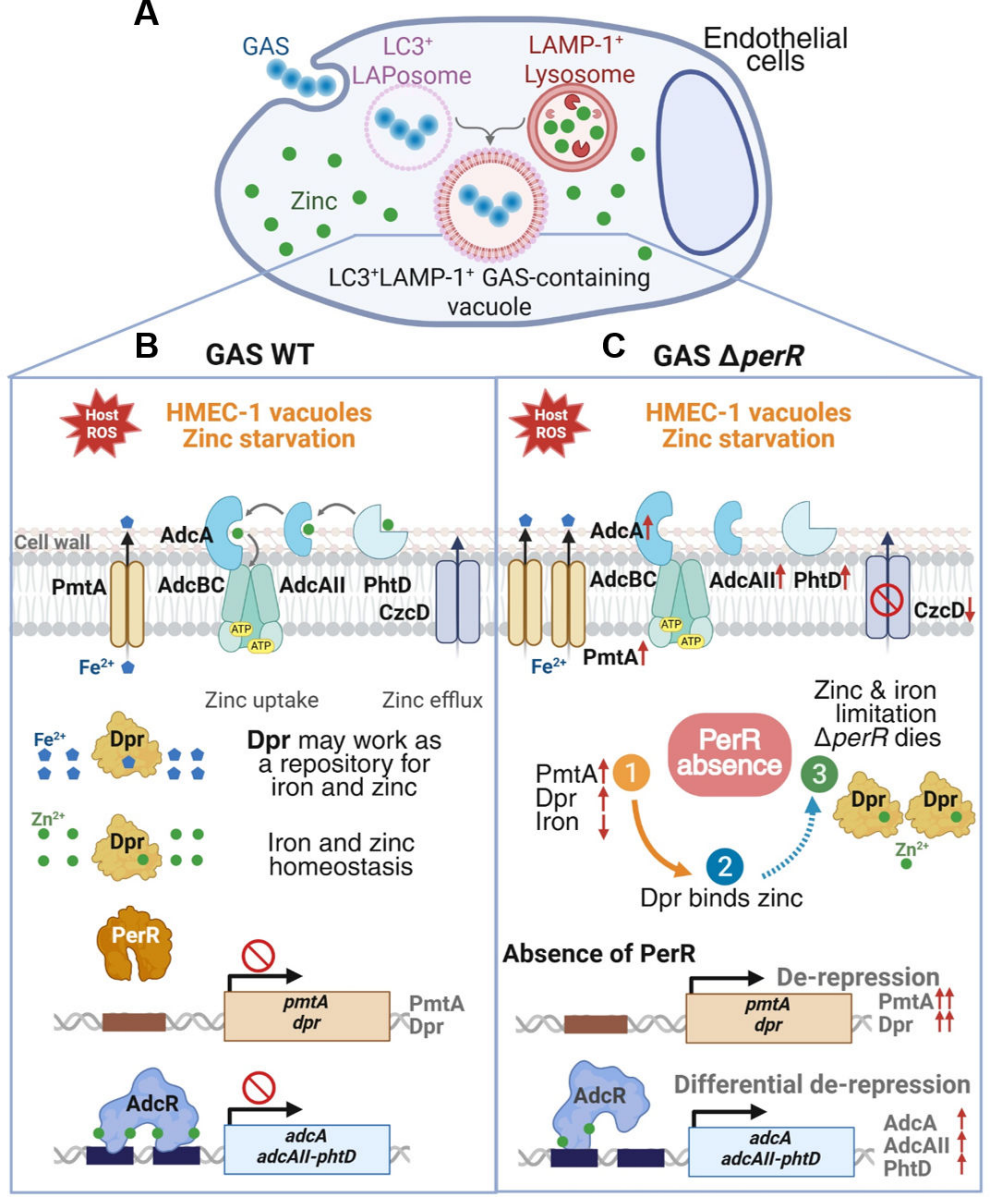
Model of PerR’s role in coordinating iron and zinc through Dpr for GAS fitness in endothelial cells. (**A**) GAS invades human endothelial cells. Invaded GAS is enclosed in LC3^+^ LAPosomes of innate immune response in endothelial cells. After fusing with LAMP-1^+^ zinc-containing lysosomes, GAS is kept inside the LC3^+^LAMP-1^+^ vacuoles, where zinc is limited. (**B**) When PerR is present in the GAS WT strain, the expression of *pmtA* and *dpr* genes is suppressed. When ROS occurs, PerR is released from the Per box. The *pmtA* and *dpr* genes are upregulated to cope with iron-mediated oxidative stress by PmtA’s iron efflux and antioxidant ferritin-like Dpr proteins, maintaining iron and zinc homeostasis. Meanwhile, the AdcR regulon is repressed due to zinc homeostasis, though microenvironmental zinc starvation occurs in the HMEC-1 vacuoles. (**C**) In the GAS Δ*perR* mutant, (1) because the PerR repressor is absent, the *pmtA* and *dpr* genes are overexpressed. Overexpressed PmtA transporters pump much iron out, leading to a low amount of intracellular iron. (2) In parallel, overexpressed iron and zinc chelating-like Dpr proteins bind to zinc, possibly causing intracellular zinc depletion. This makes the Δ*perR* mutant differentially upregulate certain zinc acquisition genes (*adcA* and *adcAII-phtD* with two AdcR motifs) of the AdcR regulon and downregulate the *czcD* gene of zinc efflux. (3) However, in the HMEC-1 cells, the zinc restriction observed inside the phagolysosome GAS-containing vacuoles may result from host zinc starvation, which severely reduces the survival of the Δ*perR* mutant. Consequently, PerR may coordinate iron and zinc through Dpr, thus enhancing GAS fitness during its invasions of human endothelial cells.

The present study provides new evidence that the PerR regulon helps GAS cope with iron and zinc homeostasis, which aids in the GAS fitness of human endothelial cells. We hypothesize that the sustainable expression of PmtA and Dpr in the Δ*perR* mutant during infection causes intracellular iron depletion and a struggle for zinc acquisition in the Δ*perR* mutant (Fig. 6). Our findings demonstrate that the GAS Δ*perR* mutant can survive the hydrogen peroxide challenge and zinc intoxication *in vitro*; however, it cannot survive when zinc is depleted or when it invades endothelial cells. Without PerR suppression, the PerR-dependent *pmtA* and *dpr* are consistently upregulated, causing increased iron efflux and reduced amounts of intracellular iron in the Δ*perR* mutant. This may force the overexpressed Dpr to acquire zinc, thus resulting in mild iron-driven zinc depletion. This subsequently causes enhanced zinc acquisition (*adcA*, *adcAII*, and *phtD*) and decreased zinc efflux (*czcD*) in the Δ*perR* mutant. We also demonstrate that the GAS WT and Δ*perR* mutant encounter zinc restriction inside the phagolysosome GAS-containing vacuoles of endothelial cells (Fig. 5A, B, and 6A). This host’s zinc starvation severely reduces the Δ*perR*’s chances of survival. Consequently, our investigation demonstrates the role GAS PerR has in coordinating iron and zinc homeostasis through PmtA’s iron efflux, iron and zinc-chelating ferritin-like Dpr proteins, and the AdcR regulon for bacterial fitness during the infection of endothelial cells.

PerR-regulated PmtA and Dpr are of importance for the modulation of oxidative stress and GAS virulence. PmtA is a ferrous iron efflux pump that enhances GAS virulence and defenses against host iron intoxication and oxidative stress (22, 23). Our GAS NZ131 Δ*dpr* mutant showed comparable CFU multiplicity as the WT strain in the HMEC-1 cells (Fig. 4A). However, we discovered the critical role of overexpressed Dpr in our Δ*perR* mutant during infection, which is contradicted by some PerR studies where *dpr* expression is not upregulated in the Δ*perR* mutants they studied (15, 25, 31). The reason for these different results is that the *dpr* promoter regions in those GAS strains were found to be highly similar in our analysis (Fig. S8). Our EMSA analysis also showed that PerR binds to the promoters of *pmtA* and *dpr* (Fig. 2H). When we re-analyzed the cDNA microarray data sets (GEO accession number: GSE6384) (25), the scanning signals of *dpr* probes were unexpectedly close to or over-saturation (Table S4). This technical bias could explain why *dpr* overexpression was neglected in the results of the previous studies. After we recalculated the unsaturated *dpr* signals, the expression of *dpr* had about a 3.3-fold increase in the HSC5 Δ*perR* mutant (Table S4), which agrees with our finding (Fig. 2C; Table S3). Our reconstructed structure of dodecameric and monomeric Dpr suggests that Dpr functions as an iron and zinc repository (Fig. S6). Furthermore, overexpressed Dpr improved the GAS growth when inoculated in higher zinc media (Fig. 4E), making GAS Dpr function like human ferritin that stores and transfers iron and zinc (40). Neither promoter analysis nor EMSA revealed Per box and PerR-*adcA* promoter binding (Fig. 2F and I). Therefore, we assume that abundant PmtA proteins pump iron out from the cytosol of the Δ*perR* mutant, and this creates a shortage of iron that leads Dpr proteins to acquire zinc. This might indirectly cause a zinc limitation for the AdcR regulon in the Δ*perR* mutant. Future investigations of the competitions for iron and zinc binding between GAS PmtA and Dpr, and attempts to discover whether iron depletion causes Dpr to bind zinc, will be important for comprehending the molecular mechanisms of GAS PerR-modulated iron and zinc homeostasis.

Nonetheless, our interpretation is supported by the study conducted by Sanson and colleagues (26). The zinc acquisition genes of GAS are differentially modulated by the zinc-bound AdcR repressor in correlation to the number of AdcR motifs in the promoter regions. When there is sufficient zinc, zinc-binding AdcR (four zinc ions) is activated and occupies the AdcR motifs. However, when there is a mild zinc shortage, partially zinc-bound AdcR alters protein conformation that then causes AdcR to occupy one motif and de-repress the other (Fig. 6B and C). Subsequently, this activates the transcription of the *adcA*, *adcAII*, and *phtD* genes whose promoter regions contain two AdcR motifs, but not the *adcRCB* and *phtY* genes (Fig. 2F) (26). Overall, our investigation explains how PerR delicately coordinates the homeostasis of iron and zinc through iron efflux exporter *pmtA* and iron- and zinc-chelating ferritin-like *dpr* to maintain GAS fitness during cell infection. However, our findings from the *in vitro* models do not reproduce the complex host-GAS interactions that occur in animal models or human subjects.

When metal ions (zinc and iron) become scarce at the sites of infection, this creates strong pressure for hosts and pathogens to develop strategies for metal acquisition and homeostasis (41). Our results show that calprotectin does not appear to be highly expressed in HMEC-1 cells, and the transcription of cellular zinc transporters and zinc-related proteins in HMEC-1 cells was not significantly altered (Table S5). This could be due to the low genome-wide coverage of our dual RNA-seq results (42). In endothelial cells, we detected the zinc signal in the phagolysosomes (LC3^+^LAMP-1^+^ vacuoles) (Fig. 5). Surprisingly, the zinc signal was almost undetectable in the LC3^+^LAMP-1^+^ vacuoles that contained either GAS WT or the Δ*perR* mutant (Fig. 5), differing from the zinc intoxication in the phagolysosomes of GAS-infected neutrophils (29, 30). In our model of the Δ*perR*-infected HMEC-1 cells (Fig. 6C), the endothelial cells sequestrate free zinc that restrains the growth of the Δ*perR* mutant, whose intracellular free zinc content is low because of overexpressed Dpr. The host zinc starvation may cause the Δ*perR* to be unable to proliferate and then to be cleared out by the host’s innate immune responses (Fig. 1E). Whether this host’s zinc starvation mechanism is part of nutritional immunity or other uncharacterized mechanisms at the post-translational level is worthy of further investigation. Collectively, with our novel finding regarding upregulated *dpr* and *pmtA* in the Δ*perR* mutant during HMEC-1 infection, the PerR-regulated iron modulation is more important than had been previously thought, and the iron- and zinc-chelating ferritin-like Dpr may play an important role in GAS fitness during cell invasions. Our findings fill a gap in our understanding of how PerR combats innate immunity and may help in the development of new anti-GAS treatments.

## Data Availability

The raw fastq data sets generated from the whole-genome sequencing analysis of the ∆*perR* mutant are deposited in the NCBI BioProject repository with accession number PRJNA1357628. The raw fastq data sets of our dual RNA-seq analysis are deposited in the NCBI Gene Expression Omnibus (GEO) repository with accession number GSE263247 (http://www.ncbi.nlm.nih.gov/geo/).

## SUPPLEMENTAL MATERIAL

Supplemental material msystems.01636-25-s0001.docx Supplemental methods and Fig. S1-S8.

Table S1 msystems.01636-25-s0002.docx List of primers.

Table S2 msystems.01636-25-s0003.xlsx WGS results.

Table S3 msystems.01636-25-s0004.docx RNAseq qPCR.

Table S4 msystems.01636-25-s0005.xls Reanalysis of cDNA microarray.

Table S5 msystems.01636-25-s0006.xls Gene expression of HMEC-1.

ASM does not own the copyrights to Supplemental Material that may be linked to, or accessed through, an article. The authors have granted ASM a non-exclusive, world-wide license to publish the Supplemental Material files. Please contact the corresponding author directly for reuse.
