# Supplementary material for: Group A streptococcal PerR coordinates iron and zinc homeostasis through Dpr, aiding in bacterial fitness during endothelial cell infection
Source: mSystems. 2026 Jan 26;11(2):e01636-25. doi: 10.1128/msystems.01636-25 (PMC12911349; doi:10.1128/msystems.01636-25)
Supplement: Supplemental material — Supplemental methods and Fig. S1-S8. [file msystems.01636-25-s0001.docx]

**Supplementary material and methods**

**Long fragment (>10 kb) genomic DNA (gDNA) isolation of Group A *Streptococcus* for whole genome sequencing (WGS) analysis.** Freshly grown overnight GAS pellets (2.5 OD_600_ per tube) were collected and resuspended in 200 μL of P1 buffer (50 mM Tris-HCl, pH 8.0; 10 mM EDTA). The resuspended cells were then transferred to a 2 mL tube containing sterile silica beads (100-150 mg per tube; 0.1 mm ZIRCONIA/SILICA, BioSpect Products). To break the cell walls, we used a bench-top vortexer for beading for 20 seconds twice. By adding 800 μL of Genomic Lysis Buffer (Zymo Research) supplemented with 0.5% beta-mercaptoethanol to the lysates, we inverted the tubes gently 20 times until the lysate mixtures formed homogeneous solutions at room temperature (RT) for about 3-5 min. Next, we transferred the supernatant of the cell lysate to a new tube, following the addition of 0.3-time (v/v) isopropanol to precipitate gDNA. To obtain gDNA, we applied the lysates to a Zymo IC Column (Zymo Research) and then centrifugation at 10,000 *g* for 2 min at RT. The washing procedures included 200 μL of W1 Buffer (Geneaid), 500 μL of Wash Buffer (Geneaid), and 500 μL of 70% ethanol. Finally, the long fragment gDNA was eluted with 20 μL of 10 mM Tris-HCl buffer (pH 8.0) or EB buffer (50 mM Tris-HCl, pH 8.5) onto the column by centrifugation (10,000 *g* for 2 min). Purified gDNA samples were stored at -20ºC until use.

**WGS library preparation and sequencing.** WGS libraries were prepared and sequenced by Welgene company (Taipei, Taiwan). In brief, we used a modified protocol based on the SureSelect XT Low Input Enrichment System (Version F0, 2022, Agilent Technologies). A total of 200 ng of gDNA was fragmented with the M220 Focused-ultrasonicator (Covaris) to a major peak size range of 350- 400 bp. The fragmented DNA was end-repaired, then ligated with TruSeq UD Indexes (Illumina). Subsequently, the adapter-ligated libraries were amplified using the SureSelect XT Low Input Reagent Kit (Agilent Technologies). Both adapter-ligated and PCR-amplified libraries were purified using AMPure XP beads (Beckman Coulter). The PCR-amplified libraries underwent size selection to obtain 350- 600 bp fragments using the BluePippin system (Sage Science). Library quality and fragment size distribution were assessed using the Agilent D1000 ScreenTape on TapeStation (Agilent Technologies). Sequencing was analysed on the NovaSeq X Series (Illumina) using the NovaSeq X Series 25B Reagent Kit (300 cycles) with a 150 bp paired-end (150PE) protocol.

**Cell cytotoxicity detection using** **lactate dehydrogenase (LDH) assay.** Cell cytotoxicity was determined by quantification of the release of cellular LDH activity from damaged HMEC-1 cells. We used the cell cytotoxicity detection kit (Roche, Sigma-Aldrich) according to the manufacturer’s instructions with some optimizations. Firstly, we determined that the optimal HMEC-1 cell concentration for the assay was 5×10^4^ cells per well for a 96-well plate. After that, we followed the GAS infection protocol (main text) to infect the HMEC-1 cells for 5 h, washed the cells twice with PBS buffer, and then added fresh M200 medium and Reaction mixture to measure the colorimetric changes at 492 nm. Cytotoxicity was ultimately calculated as a percentage as described in the manual (version 12).

**Supplementary figures**

**Figure S1**


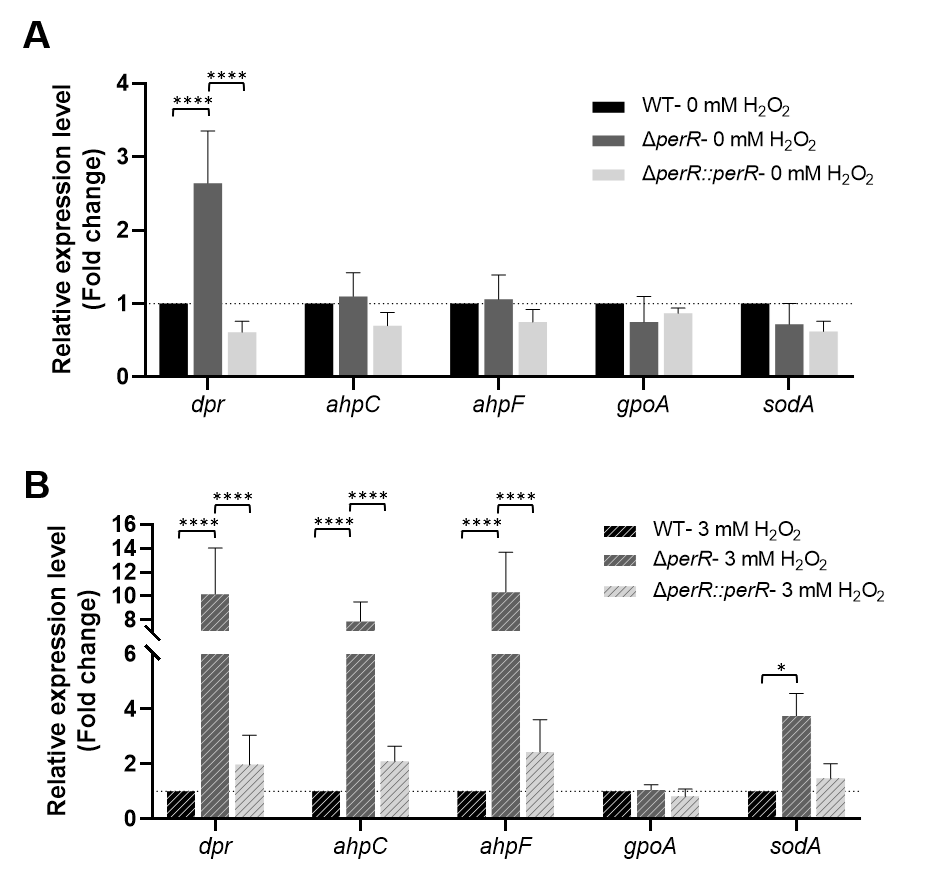


**FIG S1. Comparison of PerR-mediated ROS genes after hydrogen peroxide challenge.** Characterization of GAS ROS-related genes of PerR regulon in (A) M200 medium only (0 mM H_2_O_2_) and (B) after hydrogen peroxide challenge (3 mM H_2_O_2_) using cDNA-qPCR analysis. Expression of ROS-related genes (*dpr*, *ahpC*, *ahpF*, *gpoA*, and *sodA*) was assessed in the WT, Δ*perR*, and Δ*perR::perR* strains. Statistical significance was assessed using a two-way ANOVA with Tukey’s multiple comparisons test (*, *P* < 0.05; ****, *P* < 0.0001). All data are presented as mean values ± SD of three independent biological repeats.

**Figure S2**

**FIG S2. Cell cytotoxicity analysis using lactate dehydrogenase (LDH) assay.** Cell cytotoxicity was assessed by measuring the LDH activity from the GAS-infected HMEC-1 cells. The HMEC-1 cells were either without treatment (sample 1, mock) or infected at MOI=5 with GAS WT (sample 2), Δ*perR* (sample 3), Δ*dpr* (sample 4), as well as at MOI=50 with GAS WT (sample 5), Δ*perR* (sample 6), Δ*dpr* (sample 7) for 5 hours. Sample 8 is a positive control, as the cells were lysed using 1% Triton X-100 detergent. The higher cytotoxicity indicates more cell death and lysis. The data shown is representative of two independent biological experiments.

**Figure S3**


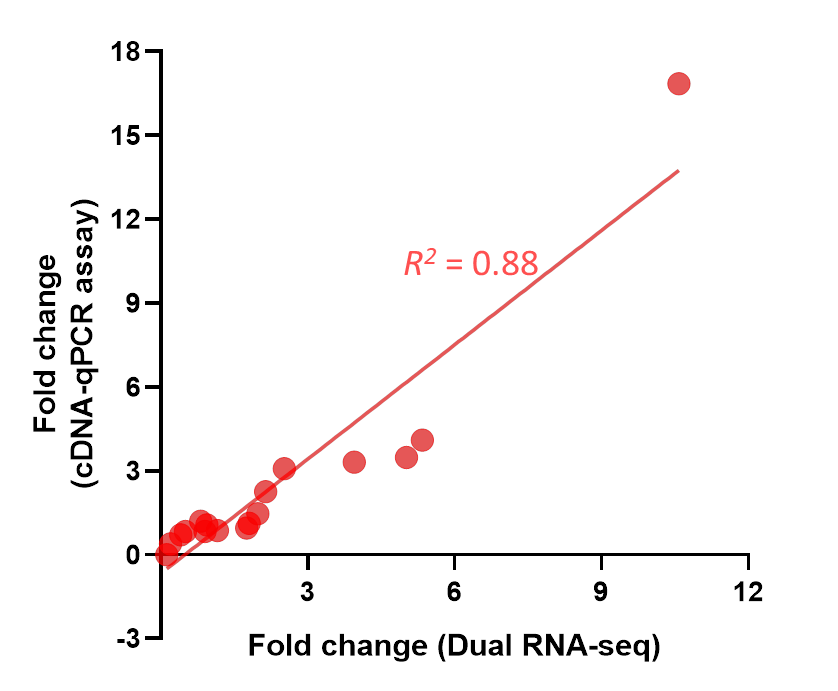


**FIG S3.** **Correlation in fold changes of the selected DEGs in the GAS Δ*perR* mutant between dual RNA-seq and cDNA-qPCR analyses.** The correlation of relative gene expression (fold change) obtained from the dual RNA-seq and cDNA-qPCR analyses is calculated and shown.

**Figure S4**

**
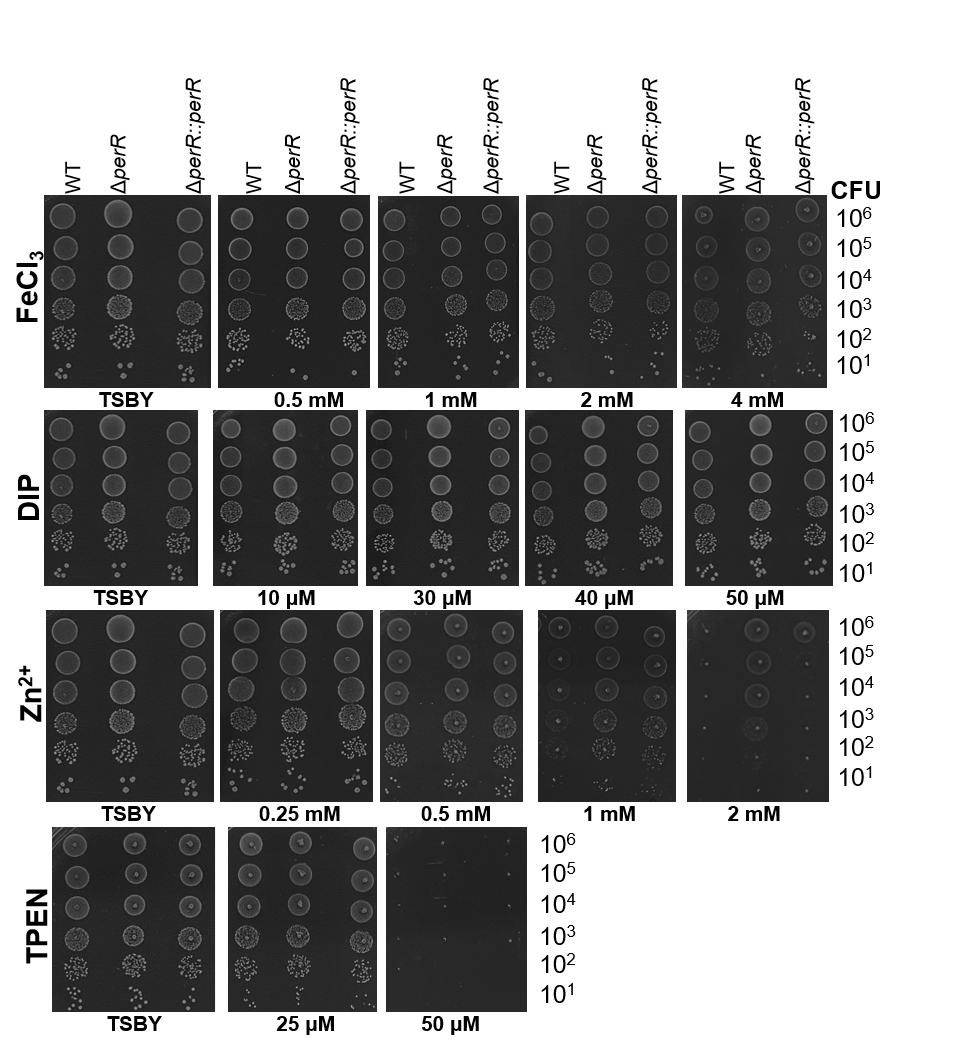
**

**FIG S4. Effect of metal ion intoxication and depletion on GAS growth using agar spot assay.** The GAS WT, Δ*perR* mutant, and Δ*perR::perR* mutant were refreshed in TSBY broth to reach mid-logarithmic phase, then deposited on the surface of TSBY agar plates supplemented with FeCl_3_ (0.5, 1, 2, and 4 mM), iron chelator DIP (10, 30, 40, 50 μM), ZnSO_4_ (0.25, 0.5, 1, and 2 mM), or zinc chelator TPEN (25 and 50 μM). The data shown is representative of four independent biological experiments

**Figure S5**

**
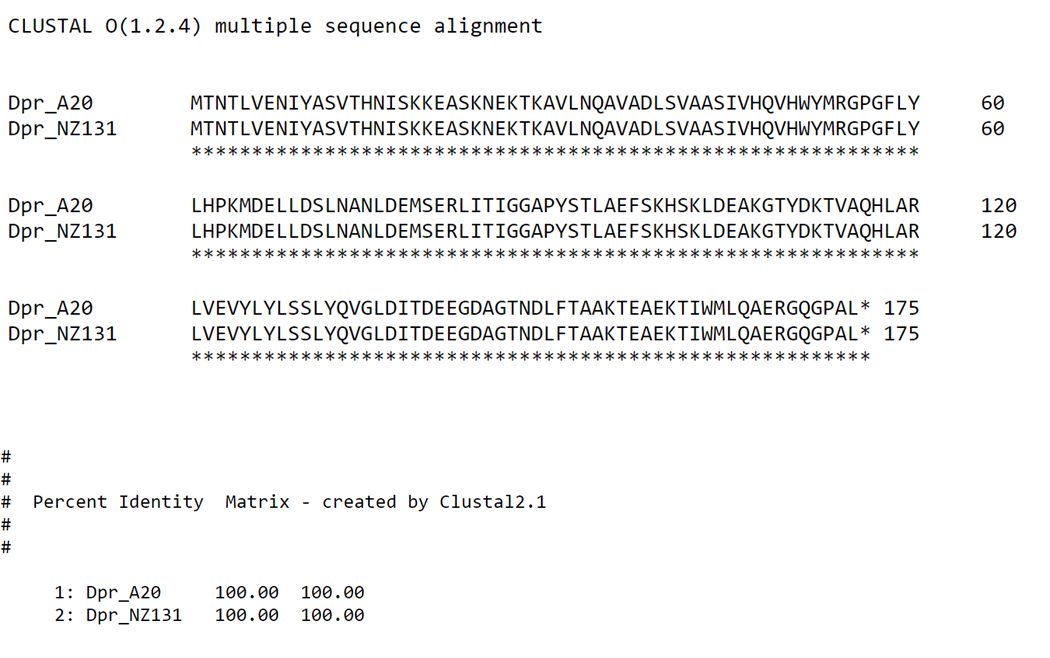
**

**FIG S5. Dpr sequence alignment of GAS strains A20 and NZ131.** The multiple sequence alignment of A20 Dpr and NZ131 Dpr proteins is executed using CLUSTAL O, and the sequence identity is 100% using Clustal 2.1 on the UniProt platform.

**Figure S6**

**
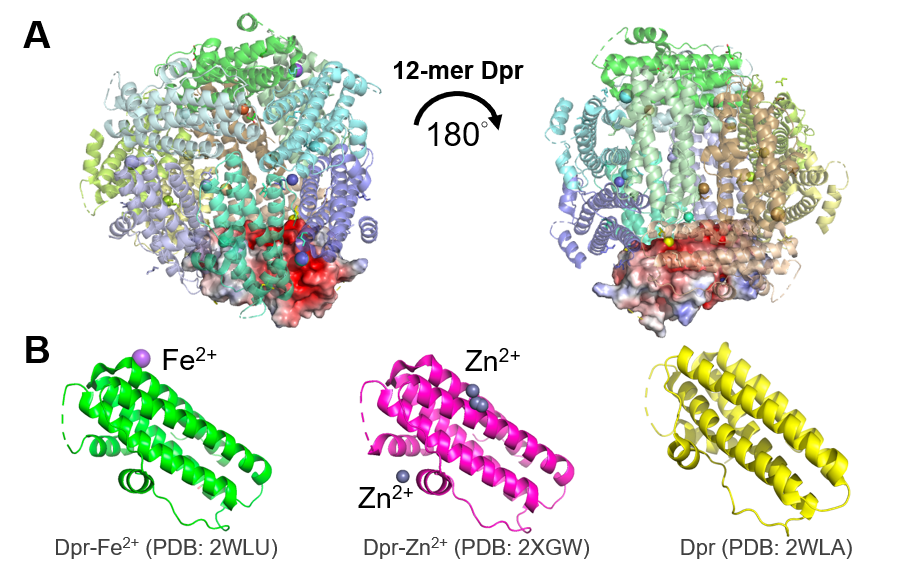
**

**FIG S6. Structural representation of assembled and monomeric Dpr.** (A) The biological assembly of Dpr. The Dpr proteins assemble into a dodecameric, spherical complex that forms a hollow, cage-like structure, with a central cavity that may serve as a repository for metal ions (PDB 2WLU). (B) Fe^2+^-bound (green; PDB 2WLU), Zn^2+^-bound (magenta; PDB 2XGW), and metal-free states of monomeric Dpr (yellow; PDB 2WLA). All three Dpr structures show similar conformation.

**Figure S7**

**
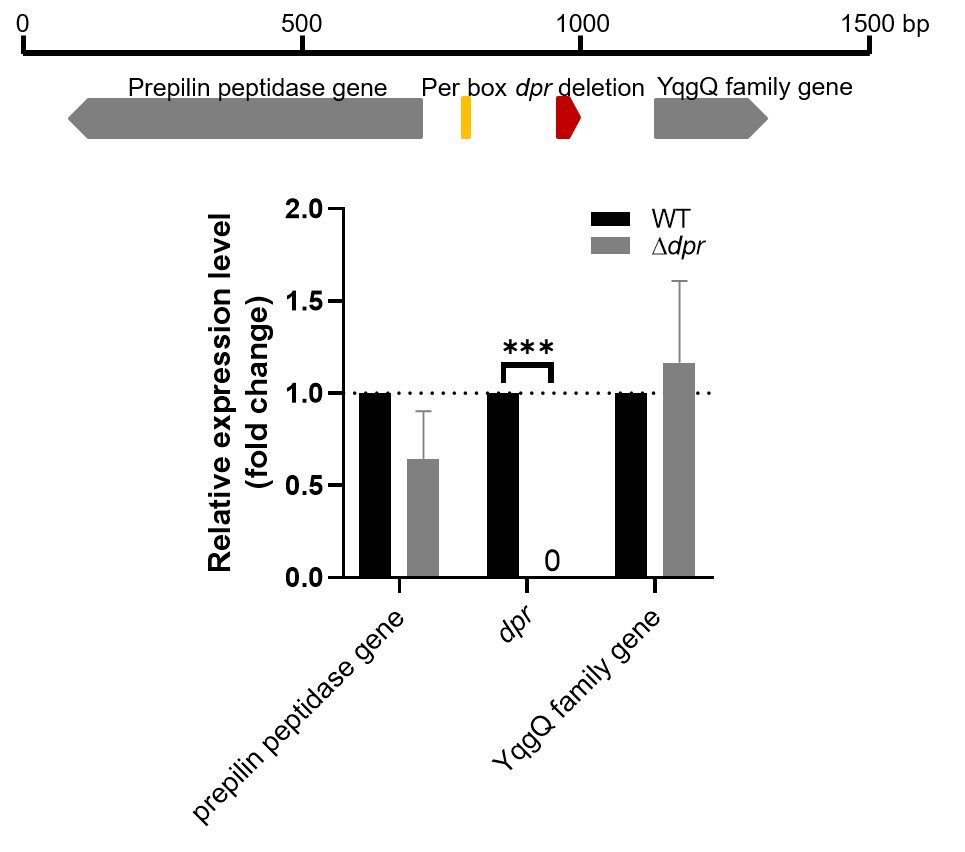
**

**FIG S7. Polarity validation of GAS Δ*dpr* mutant.** The mRNA expression of *dpr* and its up- and downstream genes was investigated in the GAS NZ131 WT and Δ*dpr* mutant using cDNA-qPCR analysis. In the Δ*dpr* mutant, the relative mRNA expression levels (fold changes) of the genes coding for prepilin peptidase and YqgQ family protein, respectively, were insignificant when compared to the WT strain. This shows that the in-frame deletion of the Δ*dpr* mutant has no polar effect. Statistical significance was assessed using a multiple *t*-test (***, *P* < 0.001). All data are presented as mean values ± SD of at least two independent biological repeats.

**Figure S8**


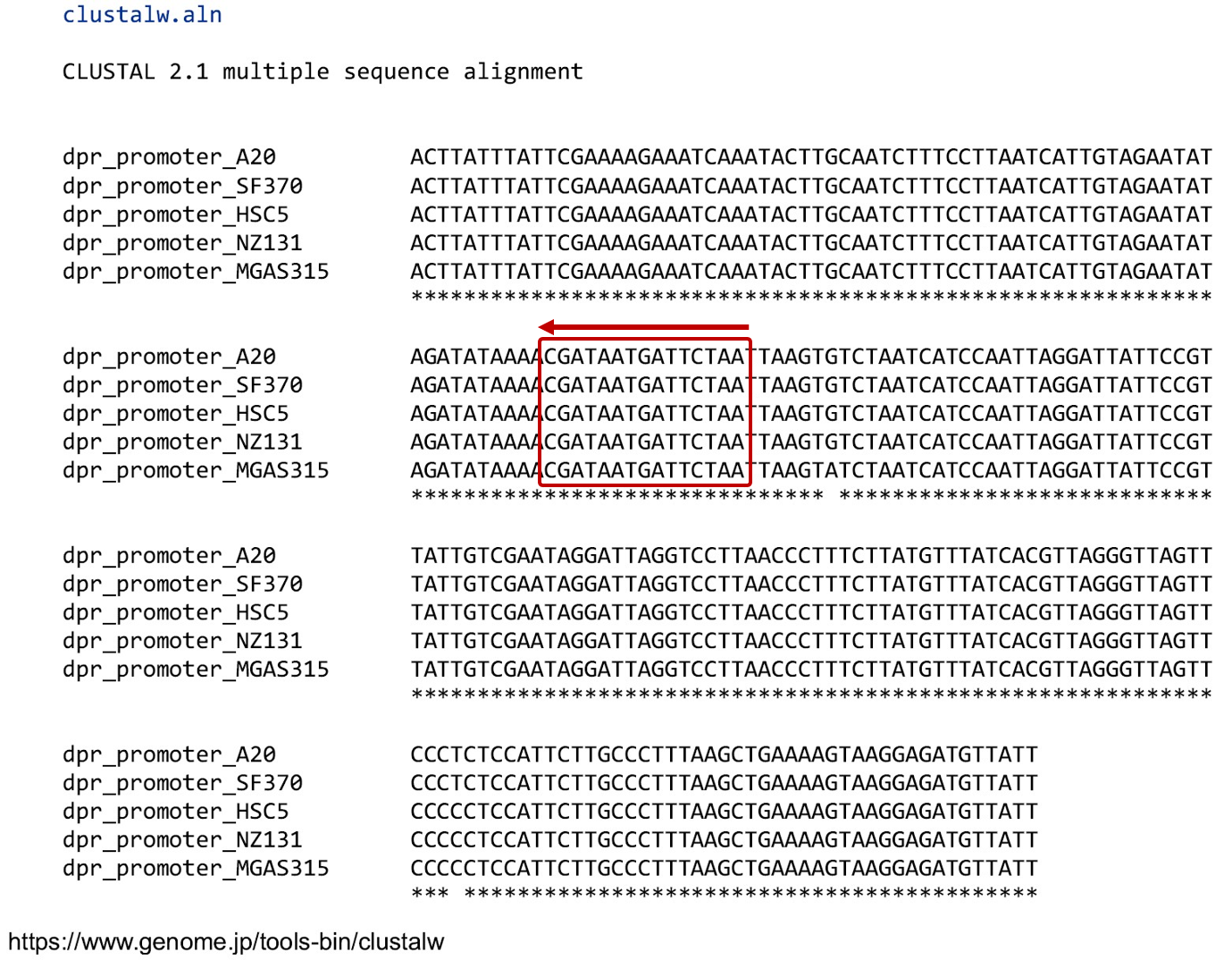


**FIG S8. Promoter region alignment of GAS *dpr* genes.** The multiple promoter sequences of GAS strains A20, SF370, HSC5, NZ131, and MGAS315 are aligned using clustalw (Clustal 2.1). The reverse Per box is labeled, and the overall DNA identity is >99%.
