# Supplementary material for: Group A streptococcal PerR coordinates iron and zinc homeostasis through Dpr, aiding in bacterial fitness during endothelial cell infection
Source: mSystems. 2026 Jan 26;11(2):e01636-25. doi: 10.1128/msystems.01636-25 (PMC12911349; doi:10.1128/msystems.01636-25)
Supplement: Table S1 — List of primers. [file msystems.01636-25-s0002.docx]

| Table S1. List of primers |  |
| --- | --- |
| **Real-time qPCR primer** | **Sequence (5’→ 3’)** |
| accD_qPCR_F5 | ACTACTGGTGGGGTAACAGCTA |
| accD_qPCR_R5 | TAACACGGCGTCCAGCAAAT |
| adcA_qPCR_F8 | GGTTGTCACCATACCGTAGCA |
| adcA_qPCR_R8 | AGCGGCATTGGCTTTGAAGT |
| adcAII/lmb_qPCR_F9 | TGAAGCGTCAAAACCTCTGACA |
| adcAII/lmb_qPCR_R9 | GCTAAAACGGGATCCGTCCA |
| adcB_qPCR_F1 | GGTAATGGCGGTAGTTGCCA |
| adcB_qPCR_R1 | CGCTACCCCAGCCAAAGAAA |
| adcC_qPCR_F9 | ACAACCAAAGGCTGGACGAG |
| adcC_qPCR_R9 | GGTGGATGGAAAACCAGCGT |
| adcR_qPCR_F8 | AGGCGGCAGTAACTAAGGCTA |
| adcR_qPCR_R8 | AAATAAGTCACCCTAGCATCAACCG |
| ahpC_qPCR_F5 | TGGTTGGCACTATCACATACCC |
| ahpC_qPCR_R5 | CACGTTGAGCAAGTCCGTCT |
| ahpF_qPCR_F2 | GGGTGACTTACTGCCCTCAC |
| ahpF_qPCR_R2 | TCCAGAGTTACCACCACCGA |
| CoA-binding protein_qPCR_F2 | TTAGCAGGTCAGGCCAAAGTC |
| CoA-binding protein_qPCR_R2 | GCAGCGGTTCATCACAATCG |
| czcD_qPCR_F2 | TGCACCATGAACTCAGCCAA |
| czcD_qPCR_R2 | AGTGGTAGATTCTTTCAAGCAGCA |
| dpr-qPCR-1 | ATGCGTGGTCCAGGTTTCTT |
| dpr-qPCR-2 | AATAAGGCGCTCCGCCAATA |
| fabK_qPCR_F10 | GTCATTGCGGCAGGTGGTAT |
| fabK_qPCR_R10 | GCAACAACAAAGCGAGTTCCA |
| gczA_qPCR_F6 | TGCGCTGCAAGTATATCGACAA |
| gczA_qPCR_R6 | TTGCCTTTGATGAAGCCACCA |
| gpoA_qPCR_F9 | CAAAACGGGAATGACCTTTCCTTAG |
| gpoA_qPCR_R9 | AATCCACACTTGGTAGCTGTGTT |
| gyrA-qPCR-1 | CCGACTGGTGCCCTTGTTAT |
| gyrA-qPCR-2 | CGTTCCCGACCTGTTTGAGT |
| mntE_qPCR_F7 | GCAAGTTCCCTTATTGCTGATGG |
| mntE_qPCR_R7 | ATGATTGGCATCGGCTGGTT |
| nga inhibitor-qPCR-F10 | TAGAGTATGGGGTTTACCCTGCT |
| nga inhibitor-qPCR-R10 | GCTTGACCGCTATTTGACATTTCT |
| nga_qPCR_F4 | TGCCAAACGTGGTCAGGAAA |
| nga_qPCR_R4 | GCCTTTGGCATCAATCTCTGC |
| nrdH_qPCR_F7 | TAGAACAACACGGAGCCAACTT |
| nrdH_qPCR_R7 | TAACAGGAGCAGATGTGAAGCC |
| perR-qPCR-1 | TGGGCCATCAACACGTCAAT |
| perR-qPCR-2 | ACCTGTTTGCTCATGGGCTT |
| phtD_qPCR_F9 | CTCAGATTGCCTTTGCCGAAC |
| phtD_qPCR_R9 | CTACAGCAAGTCGTGGCTCA |
| pmtA_qPCR_F2 | TTGGACTTGGTTGGCTGCTT |
| pmtA_qPCR_R2 | TGGCTGCACGAGAAATAGCA |
| polA_qPCR_F3 | GCCAAGGCTGTTAATTTTGGTATCG |
| polA_qPCR_R3 | AGACTTGGCCTGTTTACGGGT |
| prepilin_peptidase_qPCR_F1 | TTTTCGGCAGCTCCAAAACC |
| prepilin_peptidase_qPCR_R1 | AGCACTAACCCAATGTACCATACT |
| rpsN2_qPCR_F7 | ACCAAAAGCAGCTCCAACTGA |
| rpsN2_qPCR_R7 | GGCAGTTTGCGCAGTGATTC |
| sdaB_qPCR_F9 | AGGCTGGACTGGAAACCCT |
| sdaB_qPCR_R9 | AGTGCATCTCCACCGAGACTA |
| slo_qPCR_F5 | GTTGAAGCGGCCTTTAGTGC |
| slo_qPCR_R5 | TTGTGCTCTGCAGCATCTCC |
| sodA_qPCR_F1 | GTGGCGGACACCTAAACCAT |
| sodA_qPCR_R1 | TGGCTTGTGCAACATCAGGA |
| speB_qPCR_F3 | GTAGGCGGACATGCCTTTGT |
| speB_qPCR_R3 | AGAAGCCGTCAGAGACTCCA |
| Spy49_RS01010_qPCR_F10 | AATCGCGTCGTCGCTTGAA |
| Spy49_ RS01010_qPCR_R10 | ATGGTCAGCCTCAAGTCATCAAA |
| YqgQ_qPCR_F1 | GCGTCTCTATGATAGCGGCT |
| YqgQ_qPCR_R1 | TCTAATCTATGCTCACGTCGCA |
| **PCR primer** | **Sequence (5’→ 3’)** |
| dpr_comple_F_BamHI | CGGGATCCAATAAGATGCCCGAACATA |
| dpr_comple_R_SalI | ACGCGTCGACATAAAGACGTTTGCCAAGGT |
| dpr_del_1_BamHI | CGGGATCCCTTGATGACAAAATATTCCTAG |
| dpr_del_2*_StuI | AAGGCCTTTATGTGTTTGTCATAATAACATCTCC |
| dpr_del_3*_StuI | GAAGGCCTTGAGGACAAGGCCCAGCACTCTAA |
| dpr_del_4_BamHI | CGGGATCCCTATTTTGTGATAAATTGTCG |
| pCN143_seq_F | TCTATCCCAATTCCAAAAGG |
| pCN143_seq_R | CGCATCCGATTGCAGTAT |
| perR(spy49_0165)-PCR-1-SmaI | TCCCCCGGGTTGTAGGAGAAGTCATGAAGC |
| perR(spy49_0165)-PCR-2-BamHI | CGGGATCCTTCCGATGCTCTCACAGAT |
| perR(spy49_0165)-mut-1d*-PstI | AACTGCAGTCAGTCCATAAGCTGCTACTCCT |
| perR(spy49_0165)-mut-2-XhoI | CCGCTCGAGTCAACCTGATTTTTAGAGTTAACC |
| perR-comple-2-SalI | ACGCGTCGACGTTAACTCTAAAAATCAGGTTGATC |
| perR-comple-13-BamHI | CGGGATCCTAAACGGGCTGTTCCT |
| perR-exp-F-NdeI | GGAATTCCATATGGACATTCATTCACATCAGC |
| perR-exp-R2-XhoI | CCGCTCGAGAAAATCAGGTTGATCTTTTGC |
| **EMSA primer** | **Sequence (5’→ 3’)** |
| adcA_pro_EMSA_F1 | AGTAATCGCAAAAAGACAA |
| adcA_pro_EMSA_R1 | GACACTGATTAAACTCATC |
| adcA_pro_EMSA_R2 | CATGATTCCTCCTTTGTT |
| dpr_pro_EMSA_F2 | GTGTCTAATCATCCAATTAGG |
| dpr_pro_EMSA_R2 | CGTGATAAACATAAGAAAGGG |
| dpr_pro_EMSA_F3 | GGACCTAATCCTATTCGAC |
| dpr_pro_EMSA_R3 | TCAAATACTTGCAATCTTTCC |
| pmtA_pro_EMSA_F1 | TTGAAAGCAAAAGGTAAGTT |
| pmtA_pro_EMSA_R1 | CTCGCAATTAAAAGTTATTCAATC |
| pmtA_pro_EMSA_F3 | TGTTTATTGATAGCGACAAG |
| pmtA_pro_EMSA_R3 | GGATGACTGACCATTATAGA |
