# Supplementary material for: Group A streptococcal PerR coordinates iron and zinc homeostasis through Dpr, aiding in bacterial fitness during endothelial cell infection
Source: mSystems. 2026 Jan 26;11(2):e01636-25. doi: 10.1128/msystems.01636-25 (PMC12911349; doi:10.1128/msystems.01636-25)
Supplement: Table S3 — RNAseq qPCR. [file msystems.01636-25-s0004.docx]

Table S3. The GAS differentially expressed genes (DEGs) identified in the dual RNAseq analysis and cDNA-qPCR validation

|  |  |  |  | **RNA-seq**  **(∆*perR/*WT)** | |  | | **cDNA-qPCR (∆*perR/*WT)** | | | |
| --- | --- | --- | --- | --- | --- | --- | --- | --- | --- | --- | --- |
| **Gene locus**  **new** | **Gene locus**  **old** | **Gene ID** | **Function** | **Fold change** | ***P*adj** | |  | **Fold**  **change** | **SD** |  |  |
| **Virulence factors** | | | | | | | | | | |  |
| SPY49_RS00905 | Spy49_0144 | *nga* | Nicotine adenine dinucleotide glycohydrolase | 1.27 | 5.95E-01 | |  | n.d. | n.d. |  |  |
| SPY49_RS00915 | Spy49_0146 | *slo* | Cholesterol-dependent cytolysin streptolysin O | 1.08 | 9.19E-01 | |  | n.d. | n.d. |  |  |
| SPY49_RS08345 | Spy49_1690c | *speB* | Cysteine proteinase exotoxin | 0.71 | 8.57E-01 | |  | n.d. | n.d. |  |  |
| SPY49_RS08355 | Spy49_1692c | *sdaB* | Streptodornase B | 2.52 | 8.09E-11 | |  | 3.07 | 0.58 |  |  |
| **ROS regulation** | | | | | | | | | | |  |
| SPY49_RS01005 | Spy49_0165 | *perR* | Peroxide-responsive transcriptional repressor | 0.12 | 2.74E-35 | |  | 0.00 | 0.00 |  |  |
| SPY49_RS05850 | Spy49_1183c | *dpr* | Dps-like peroxide resistance protein, non-specific DNA-binding/iron-binding ferritin-like antioxidant protein | 2.14 | 2.84E-20 | |  | 2.25 | 0.42 |  |  |
| SPY49_RS05665 | Spy49_1146c | *pmtA* | Heavy metal translocating P_1B-4_-type ATPase | 10.58 | 3.35E-117 | |  | 16.85 | 4.20 |  |  |
| SPY49_RS08490 | Spy49_1721 | *ahpC* | Peroxiredoxin; alkyl hydroperoxide reductase subunit C | 1.22 | 3.30E-01 | |  | n.d. | n.d. |  |  |
| SPY49_RS08495 | Spy49_1722 | *ahpF* | Alkyl hydroperoxide reductase subunit F | 1.17 | 4.57E-01 | |  | n.d. | n.d. |  |  |
| SPY49_RS02670 | Spy49_0511c | *gpoA* | Glutathione peroxidase | 0.91 | 9.17E-01 | |  | n.d. | n.d. |  |  |
| SPY49_RS05565 | Spy49_1122c | *sodA* | Superoxide dismutase | 1.12 | 5.78E-01 | |  | n.d. | n.d. |  |  |
| **Zinc homeostasis** | | | | | | | | | | |  |
| SPY49_RS00600 | Spy49_0080 | *adcR* | MarR family transcriptional regulator | 1.16 | 7.04E-01 | |  | 0.87 | 0.17 |  |  |
| SPY49_RS00605 | Spy49_0081 | *adcC* | Zinc metal ABC transporter ATP-binding protein | 0.93 | 8.80E-01 | |  | 1.06 | 0.12 |  |  |
| SPY49_RS00610 | Spy49_0082 | *adcB* | Zinc ABC transporter, permease protein | 0.81 | 4.98E-01 | |  | 1.20 | 0.32 |  |  |
| SPY49_RS02860 | Spy49_0549 | *adcA* | Zinc ABC transporter substrate-binding protein | 3.95 | 1.41E-29 | |  | 3.31 | 0.56 |  |  |
| SPY49_RS08240 | Spy49_1667c | *lsp/lmb/adcAII* | Metal ABC transporter substrate-binding lipoprotein/laminin-binding adhesin | 5.34 | 6.37E-12 | |  | 4.10 | 0.87 |  |  |
| SPY49_RS08235 | Spy49_1666c | *phtD* | Pneumococcal-type histidine triad protein PhtD | 5.01 | 5.02E-17 | |  | 3.48 | 1.05 |  |  |
| SPY49_RS07605 | Spy49_1544 | *rpsN2* | 30S ribosomal protein S14 | 1.80 | 1.36E-02 | |  | 1.12 | 0.34 |  |  |
| SPY49_RS05385 | Spy49_1084c | *phtY* | Pneumococcal-type histidine triad protein PhtY | 1.75 | 2.34E-01 | |  | n.d. | n.d. |  |  |
| SPY49_RS03400 | Spy49_0664 | *gczA* | TetR/AcrR family transcriptional regulator | 1.75 | 3.99E-01 | |  | 0.96 | 0.10 |  |  |
| SPY49_RS03395 | Spy49_0663c | *czcD* | Zinc cation transporter | 0.19 | 5.14E-02 | |  | 0.39 | 0.11 |  |  |
| **Others** | | | | | | | | | | |  |
| SPY49_RS02940 | Spy49_0566c | *Protein coding* | Helix-turn-helix domain-containing protein; regulatory protein-RofA related | 1.98 | 1.13E-03 | |  | 1.46 | 0.16 |  |  |
| SPY49_RS03075 | Spy49_0596 | *pheS* | phenylalanine-tRNA ligase subunit alpha | 0.48 | 3.1E-02 | |  | n.d. | n.d. |  |  |
| SPY49_RS03330 | Spy49_0650 | *pyrR* | bifunctional pyr operon transcriptional regulator/uracil phosphoribosyltransferase | 0.50 | 2.7E-03 | |  | n.d. | n.d. |  |  |
| SPY49_RS05040 | Spy49_1009 | *mntE* | Cation diffusion facilitator family transporter | 0.90 | 8.48E-01 | |  | 0.83 | 0.12 |  |  |
| SPY49_RS05445 | Spy49_1096 | *nrdH* | Glutaredoxin-like protein | 0.40 | 6.65E-03 | |  | 0.67 | 0.09 |  |  |
| SPY49_RS06725 | Spy49_1359c | *Protein coding* | acetyl-CoA carboxylase carboxyl transferase subunit alpha | 0.48 | 1.0E-06 | |  | n.d. | n.d. |  |  |
| SPY49_RS06730 | Spy49_1360c | *accD* | Acetyl-CoA carboxylase, carboxyltransferase subunit beta | 0.50 | 2.08E-06 | |  | 0.82 | 0.25 |  |  |
| SPY49_RS06735 | Spy49_1361c | *Protein coding* | acetyl-CoA carboxylase biotin carboxylase subunit | 0.44 | 3.8E-06 | |  | n.d. | n.d. |  |  |
| SPY49_RS06740 | Spy49_1362c | *fabZ* | 3-hydroxyacyl-ACP dehydratase | 0.43 | 1.2E-06 | |  | n.d. | n.d. |  |  |
| SPY49_RS06750 | Spy49_1364c | *fabF* | beta-ketoacyl-ACP synthase II | 0.45 | 7.1E-05 | |  | n.d. | n.d. |  |  |
| SPY49_RS06755 | Spy49_1365c | *fabG* | 3-oxoacyl-[acyl-carrier-protein] reductase | 0.46 | 4.3E-04 | |  | n.d. | n.d. |  |  |
| SPY49_RS06760 | Spy49_1366c | *fabD* | ACP S-malonyltransferase | 0.49 | 4.6E-04 | |  | n.d. | n.d. |  |  |
| SPY49_RS06765 | Spy49_1367c | *fabK* | Enoyl-[acyl-carrier-protein] reductase | 0.40 | 2.70E-07 | |  | 0.71 | 0.16 |  |  |
| SPY49_RS07000 | Spy49_1416 | *Protein coding* | glycoside hydrolase family 32 protein | 2.34 | 1.4E-02 | |  | n.d. | n.d. |  |  |
| SPY49_RS07230 | Spy49_1464c | *Protein coding* | gp58-like family protein | 4.59 | 3.7E-03 | |  | n.d. | n.d. |  |  |
| SPY49_RS07240 | Spy49_1466c | *Protein coding* | phage tail spike protein | 4.35 | 8.3E-03 | |  | n.d. | n.d. |  |  |

n.d., not determined; highlighted genes in yellow, up-regulated DEGs in the ∆*perR* mutant from the dual RNA-seq analysis; highlighted genes in blue, down-regulated DEGs in the ∆*perR* mutant from the dual RNA-seq analysis.
